# Supplementary material for: Does peripheral neuroinflammation predict chronicity following whiplash injury? Protocol for a prospective cohort study
Source: BMJ Open. 2022 Dec 14;12(12):e066021. doi: 10.1136/bmjopen-2022-066021 (PMC9756191; doi:10.1136/bmjopen-2022-066021)
Supplement: Author's manuscript [file bmjopen-2022-066021.draft_revisions.pdf]

BMJ Open

BMJ Open is committed to open peer review. As part of this commitment we make the peer review history of every article we publish publicly available.

When an article is published we post the peer reviewers' comments and the authors' responses online. We also post the versions of the paper that were used during peer review. These are the versions that the peer review comments apply to.

The versions of the paper that follow are the versions that were submitted during the peer review process. They are not the versions of record or the final published versions. They should not be cited or distributed as the published version of this manuscript.

BMJ Open is an open access journal and the full, final, typeset and author-corrected version of record of the manuscript is available on our site with no access controls, subscription charges or pay-per-view fees (http://bmjopen.bmj.com).

If you have any questions on BMJ Open's open peer review process please email info.bmjopen@bmj.com

BMJ Open

Does peripheral neuroinflammation predict chronicity following whiplash injury? Protocol for a prospective cohort study

Journal:	*BMJ Open*
Manuscript ID	bmjopen-2022-066021
Article Type:	Protocol
Date Submitted by the Author:	24-Jun-2022

Complete List of Authors:	Ridehalgh, Colette; University of Brighton, School of Sport and Health Sciences; Brighton and Sussex Medical School, Neuroscience<br>Fundaun, Joel; University of Oxford, Nuffied Department of Clinical Neurosciences<br>Bremner, Stephen; Brighton and Sussex Medical School, Department of Primary Care & Public Health<br>Cercignani, Mara; Cardiff University, Cardiff University Brain Research Imaging Centre<br>Young, Rupert; University of Sussex School of Engineering and Informatics<br>Trivedy, Chetan; Brighton and Sussex University Hospitals NHS Trust; Queen Mary University of London<br>Novak, Alex; Oxford University Hospitals NHS Foundation Trust, Emergency Medicine Research Oxford<br>Greening, Jane; Brighton and Sussex Medical School, Neuroscience<br>Schmid, Annina ; University of Oxford, Nuffield Department of Clinical Neurosciences<br>Dilley, A.; Brighton and Sussex Medical School, Neuroscience

Keywords:	Neuromuscular disease < NEUROLOGY, Neuroradiology < NEUROLOGY, CLINICAL PHYSIOLOGY, Musculoskeletal disorders < ORTHOPAEDIC & TRAUMA SURGERY

SCHOLARONE™
Manuscripts

Does peripheral neuroinflammation predict chronicity following whiplash injury? Protocol for a prospective cohort study

Colette Ridehalgh, Joel Fundaun, Stephen Bremner, Mara Cercignani, Rupert Young, Chet Trivedy, Alex Novak, Jane Greening, Annina Schmid, Andrew Dilley

Corresponding author Colette Ridehalgh, School of Sport and Health Sciences, University of Brighton, 49 Darley Rd, Eastbourne, BN20 7UR, United Kingdom cr19@brighton.ac.uk

Department of Neuroscience, Brighton and Sussex Medical School, Trafford Centre, University of Sussex, Falmer, Brighton BN1 9RYUnited Kingdom.

Joel Fundaun (joint first author), Nuffield Department of Clinical Neurosciences, University of Oxford, Level 6, West Wing, John Radcliffe Hospital, Oxford OX3 9DU, United Kingdom

Stephen Bremner, Department of Primary Care & Public Health, Brighton and Sussex Medical School, Watson Building, University of Brighton Falmer Campus, Brighton BN1 9PH, United Kingdom

Mara Cercignani, Cardiff University Brain Research Imaging Centre, Cardiff University, CF24 4HQ, United Kingdom

Rupert Young, School of Engineering and Informatics, University of Sussex, Sussex House, Falmer

Brighton, BN1 9RH, United Kingdom

Chet Trivedy, Emergency Department, University Hospitals Sussex NHS Foundation Trust, Princess Royal Hospital, Lewes Road, Haywards Heath, RH16 4EX, United Kingdom

Queen Mary University of London, Mile End Road, London E1 4NS, United Kingdom

Alex Novak, Emergency Medicine Research Oxford (EMROx), Oxford University Hospitals NHS Foundation Trust, Oxford, OX3 9DU, United Kingdom

Jane Greening, Department of Neuroscience, Brighton and Sussex Medical School, Trafford Centre, University of Sussex, Falmer, Brighton BN1 9RY, United Kingdom

Annina Schmid (joint last author), Nuffield Department of Clinical Neurosciences, University of Oxford, Level 6, West Wing, John Radcliffe Hospital, Oxford OX3 9DU, United Kingdom

Andrew Dilley, Department of Neuroscience, Brighton and Sussex Medical School, Trafford Centre, University of Sussex, Falmer, Brighton BN1 9RY, United Kingdom

For peer review only - http://bmjopen.bmj.com/site/about/guidelines.xhtml

Word count: 3904

Keywords: Whiplash injuries; neuritis; magnetic resonance imaging; peripheral nerve injuries

For peer review only

For peer review only - http://bmjopen.bmj.com/site/about/guidelines.xhtml

ABSTRACT

Introduction

Whiplash associated disorder grade 2 (WAD2) is characterised by musculoskeletal pain and tenderness but no apparent nerve injury. However, studies have found clinical features indicative of neuropathy and neuropathic pain. Such findings may indicate peripheral nerve inflammation, since preclinical neuritis models found that inflamed, intact nociceptors become mechanically sensitive. The primary aim of this study is to establish the contribution of peripheral neuroinflammation to WAD2 and its role in prognosis. Participants recruited will be invited to participate in a sub-study investigating the contribution of cutaneous small fibre pathology to WAD2.

Methods and analysis

115 participants within one month following whiplash injury and 34 healthy control participants will be recruited and complete validated questionnaires for pain, function, and psychological factors. Clinical examination and quantitative sensory testing will be performed, and blood samples taken to establish levels of inflammatory mediators. MRI scans using T2-weighted and diffusion tensor images of brachial plexus and wrist will determine nerve inflammation and nerve structural changes. Skin biopsies from participants in the sub-study will determine structural integrity of dermal and intraepidermal nerve fibres. At six months, we will evaluate recovery using the Neck Disability Index and a self-rated global recovery question and repeat the outcome measures.

Regression analysis will be used to identify differences in MRI parameters, clinical tests and skin biopsies between WAD2 participants and age/gender-matched controls. Linear and logistic regression analyses will assess if nerve inflammation (MRI parameters) predicts poor outcome. Mixed effects modelling will compare MRI and clinical measures between recovered and non-recovered participants with whiplash over time.

Ethics and dissemination

Ethical approval has been received from London-Brighton & Sussex Research Ethics Committee (20/PR/0625) and South Central–Oxford C Ethics Committee (18/SC/0263). Results will be disseminated through publications in peer-reviewed journals, presentations at national and international conferences and through social media.

For peer review only - http://bmjopen.bmj.com/site/about/guidelines.xhtml

ARTICLE SUMMARY

Strengths and limitations

- This is the first study to evaluate the contribution of peripheral nerve inflammation to WAD2 and its role in prognosis and establish if bedside clinical tests can be used to identify these mechanisms in people after WAD2.
- The study is being carried out at two sites, which benefits the study for several reasons: Firstly, it enhances recruitment, ensuring that the sample size is met. Secondly, the results are more representative of the UK population enabling better extrapolation of the results. Finally, collaboration between the two sites enhances the quality of the study, since knowledge and expertise are shared across sites.
- Two site studies have some challenges such as potential differences in data collection across sites, but regular site visits and frequent meetings will ensure less variability between the two data sets. Additionally, identical MRI scanners are being used across the 2 sites and since healthy controls are also being scanned at both sites this enables any variation between scanners to be controlled and statistically adjusted for at the analysis stage (1).

INTRODUCTION

Whiplash injury sustained by a motor vehicle collision can cause significant long-term pain and disability. In the UK, economic costs are as high as £3 billion per year mostly due to healthcare costs (1). Most whiplash cases are considered whiplash associated disorder grade 2 (WAD2), as defined by the Quebec Task Force Classification system (2). WAD2 refers to musculoskeletal pain and tenderness but with no frank nerve injury identified during routine clinical testing (e.g., electrodiagnostic testing, neurological bedside testing). Approximately 50% of people with WAD2 develop chronic symptoms that frequently persist for more than five years (3, 4). Although a limited number of prognostic factors have been identified (5), they do not indicate a site of tissue pathology. This lack of understanding of the underlying pathophysiology likely explains why treatments for this condition are relatively ineffective (6, 7). Despite the absence of a detectable nerve injury on routine clinical testing, many of the clinical features of WAD2 indicate a syndrome that is neuropathic in nature, such as reduced vibration thresholds, cutaneous thermal hypoaesthesia and cutaneous hypersensitivities to tactile and thermal stimuli distal to the site of injury (3, 8-14). Furthermore, clinical tests of nerve trunk mechanical sensitivity, such as palpating over upper limb peripheral nerve trunks and positioning the upper limb in postures that increases the tension in the brachial plexus and median nerve (the upper limb neurodynamic test-1; ULNT1), provoke symptoms in many

For peer review only - http://bmjopen.bmj.com/site/about/guidelines.xhtml

patients (8, 9, 14). Based on these findings, we propose that some people diagnosed with WAD2 may have an undetected nerve pathology, such as peripheral neuroinflammation, which could be a cause of chronicity. Consistent with a role for neuroinflammation, animals with a localised neuritis develop tactile and thermal-evoked cutaneous hypersensitivities in the absence of axonal degeneration (15-17). Of note, intact nociceptive axons become mechanically sensitive responding to pressure and stretch (17-20), similar to the neural mechanosensitivity observed in people with WAD2.

Magnetic resonance (MR) neurography has gained increasing interest in the diagnosis of peripheral nerve pathology (21-23). An increased signal intensity on T2-weighted sequences is considered an imaging correlate of the intraneural oedema associated with peripheral neuroinflammation. Accordingly, T2-weighted signal is increased in animal models of neuroinflammation (24, 25), and correlates positively with histological findings of inflammation (25). Using T2-weighted MR neurography in a group of patients with chronic WAD2, we have identified increased signal intensity in the cervical nerve roots and the median nerve at the carpal tunnel, indicative of peripheral neuroinflammation (9). In addition to T2-weighted MR neurography, diffusion tensor imaging (DTI) has recently been employed to examine the microstructural integrity of peripheral nerves (26, 27). DTI evaluates the diffusivity of water molecules within nerves, which can provide information on the integrity of axons and myelin sheaths (28, 29), as well as intraneural oedema (30).

In addition to neuroinflammation and functional changes to the nervous system, there is also some evidence for the presence of structural nerve pathology in patients with WAD2. This is apparent by a decreased intraepidermal nerve fibre density in the index finger in patients with chronic WAD2 (14). It currently remains unknown whether such small fibre degeneration is already apparent in the early stages after injury and whether it predicts outcome.

The main aim of this study is to establish the contribution of peripheral nerve pathology to WAD2 and its role in prognosis. Our primary focus is on peripheral neuroinflammation, which we will evaluate using a detailed examination, including MRI of the brachial plexus and distal nerve trunks, blood markers and clinical tests of nerve trunk mechanical sensitivity. Additionally, recruited participants will be invited to participate in a sub-study that will use skin biopsies to establish the contribution of cutaneous nerve fibre pathology to WAD2. Our results will advance our understanding of the pathology underlying WAD2 and may identify factors that predict the development of persistent symptoms.

For peer review only - http://bmjopen.bmj.com/site/about/guidelines.xhtml

METHODS AND ANALYSIS

Objectives

The first objective of this study will determine the presence of peripheral neuroinflammation during the acute stage following a WAD2 injury. This objective will use MRI to assess the extent of peripheral neuroinflammation and nerve microstructure in a cross-section of patients within 4 weeks of injury and healthy controls. The second objective will determine whether the presence of peripheral neuroinflammation in the acute stage can predict patient recovery status at six months. The third objective will determine the course of peripheral neuroinflammation from the acute to chronic stages of a WAD2 injury. For this objective, we will examine changes in MRI measures and clinical tests from baseline to six months in both recovered and non-recovered patients. The fourth objective will determine whether low-cost clinical tests can serve as markers to detect neuroinflammation as determined on MRI. The objective of the sub-study is to determine whether the structural integrity of cutaneous nerve fibres is altered in the acute stage following a WAD2 injury, and whether it contributes to the progression to chronicity.

Study design

This is a prospective cohort study at two centres. A total of 115 participants will be recruited within one month following a whiplash injury. We will also recruit 34 healthy proportionally age- and gender-matched control participants. People with acute WAD2 and healthy control participants will attend a baseline appointment. In addition to MRI, a detailed musculoskeletal assessment will be performed, as well as tests of nerve trunk mechanical sensitivity and quantitative sensory testing (QST). A blood sample will be taken and analysed for levels of inflammatory markers. Participants will also be asked to complete a series of validated questionnaires to assess neuropathic symptoms, functional deficits, quality of life and psychological factors. For the sub-study, skin biopsies from the index finger and ankle will also be taken. At six months, the Neck Disability Index and a self-rated global recovery question will be used to determine recovery. A sequential sample of patients will be invited to attend a follow-up appointment during which the same detailed assessment will be repeated (Figure 1).

For peer review only - http://bmjopen.bmj.com/site/about/guidelines.xhtml

Fig 1. Flow diagram showing the study design.

Patient and public involvement

Patient partners were involved in the design of the study and highlighted the relevance and need for this study. They will continue to be an integral part of the research team, offering advice from conducting the study through to interpretation of the data and dissemination of findings.

Participant recruitment

Patients aged 18-60 that meet the Quebec Task Force Classification of WAD2 will be recruited within four weeks of injury. Participants will be recruited from emergency departments and minor injury units in Surrey, Sussex, Kent and Oxfordshire directly by clinicians, and following weekly hospital database searches using defined search terms (e.g., motor vehicle collision, muscle injury: neck, bruise / contusion / abrasion: neck, sprain / ligament injury: cervical spine). Potential participants will be contacted in person, or by phone, and asked for permission for their details to be passed on to the study team, and/or sent the study details. In addition, posters will be displayed in departments with study contact details. Thirty-four healthy age- and gender-matched control participants will be recruited through departmental emailing lists (Universities of Sussex and Oxford as well as local hospitals), social media and flyers on public notice boards. Potential participants will be screened for suitability via telephone and also be invited to take part in the sub study. Eligible individuals will be sent a participant information sheet and questionnaires to complete (see below), and will be invited to attend a baseline appointment at either Brighton and Sussex Medical School, University of Sussex, Falmer, or the Nuffield Department of Clinical Neurosciences, Oxford University.

Exclusion criteria include pregnancy, recent history of cervical/arm pain lasting >3 months, previous diagnosis of a peripheral neuropathy, history of systemic illness or autoimmune disease, non-medically controlled hypertension, ongoing steroid treatment. Participants will be assessed for their suitability to undergo MRI.

Both recruitment methods and experimental design, in particular length of time of research appointments and MRI scanning, have been discussed and modified based on the comments received from our patient advisory group.

For peer review only - http://bmjopen.bmj.com/site/about/guidelines.xhtml

Baseline and follow-up appointment

Written informed consent will be obtained by a member of the study team at the baseline appointment. The following procedures will be carried out (see table 1 for full details):

Table 1 Summary of self-reported and physical assessments

Test	Description	Type of tool/assessment	Participants
**Demographics**	Age, gender, height/weight	Self-reported	HC and whiplash
**Injury details**	Impact, anticipation	Self-reported	Whiplash
**Medication details**	Specific to WAD symptoms	Self-reported	Whiplash
**Symptom history**	Description of pain or symptoms	Self-reported	Whiplash
**Pain level**	Current pain severity	Visual analogue scale	Whiplash
**Management**	Any investigations/treatment	Self-reported	Whiplash
**Type of pain**	Neuropathic Pain	pain DETECT Questionnaire	Whiplash
**Disability**	Neck disability	Neck Disability Index	Whiplash
**Psychological factors**	Posttraumatic stress<br>Pain catastrophising<br><br>Depression, anxiety and stress<br>Quality of life	PTSD-8<br>Pain Catastrophising Scale<br>DASS 42<br><br>EQ-5D-5L	Whiplash
**Active range of motion**	Cervical spine<br>Glenohumeral joints<br>Elbow, wrist and hands	0-3 scale (0 =full range no symptoms, 1 full range, symptoms, 2 reduced range, no symptoms, 3 reduced range, symptoms)	HC and whiplash
**Standardised neurological examination**	Sensation (dermatomes C5-T1)<br>Myotomes (C5-T1)<br><br>Reflexes (biceps, triceps)	Cotton wool (light touch)<br>Neurotip (pin prick)<br>Isometric muscle contraction<br>Reflex hammer	HC and whiplash
**Bedside sensory testing**	Sensation- main pain area and index finger (median nerve/C6 innervation)	Warm/cold coin (thermal) and Neurotip (pinprick)	HC and whiplash
**Quantitative sensory testing- index finger**	Warm/cold sensation/pain thresholds<br>Mechanical detection thresholds<br><br>Mechanical pain thresholds	MSA thermal stimulator (Somedic, Stockholm, Sweden)<br>Von Frey filaments (Marstock NerveTest filaments<br>(MRCsystems, Heidelberg, Germany	HC and whiplash

For peer review only - http://bmjopen.bmj.com/site/about/guidelines.xhtml

		Weighted pin prick stimulators (MRC systems, Heidelberg, Germany)<br>Vibration (Tuning fork)<br>Pressure pain threshold (Algometer, Wagner Force dial, Greenwich, USA)	
**Heightened nerve mechanosensitivity**	Upper limb neurodynamic test 1 (median nerve bias)	Positive/negative test (symptom response changes with structural differentiation)<br>Inclinometer (ROM degrees)	HC and whiplash
	Upper limb neurodynamic test 3 (ulnar nerve bias)	Positive/negative test (symptom response changes with structural differentiation)	
	Nerve palpation<br>Brachial plexus<br>Median nerve proximal to carpal tunnel<br>Ulnar nerve (Guyon's canal)	Digital palpation (0-3: 0 = No pain or discomfort<br>1= local discomfort<br>2a= local painful response<br>2b=referred pain/symptoms	
	Ulnar nerve (proximal to cubital tunnel)<br>Median nerve (carpal tunnel)	Algometer and score (0-3)	
	Phalens<br>Tinels	Digital palpation (0-1)	
**Magnetic Resonance Imaging**	Brachial plexus<br>Wrist	T2 weighted images<br>Diffusion tensor images	HC and whiplash
**Serum samples**	venepuncture	inflammatory markers	HC and whiplash
**Skin samples**	Skin biopsy	Density of dermal and intraepidermal nerve fibres using immunohistochemistry	HC and whiplash

Key: WAD whiplash associated disorder; HC healthy controls; ROM range of motion; PTSD posttraumatic stress disorder; DASS depression, anxiety and stress sub-scale

For peer review only - http://bmjopen.bmj.com/site/about/guidelines.xhtml

Questionnaires

Patients will complete the following validated questionnaires: Neck Disability Index (NDI) (31, 32), pain detect questionnaire (painDETECT) (33), short post-traumatic stress inventory (PTSD8) (34), pain catastrophising scale (PCS) (35), depression, anxiety and stress scale (DASS42) (36), the quality of life questionnaire (EQ-5D-5L) (37), and the Impact of Events Scale (IES-Revised) (38).

Clinical assessment

Demographic information and type of neck injury sustained will be obtained (i.e., speed of impact, direction of collision, and initial symptoms). A body chart and visual analogue scale (0-100 mm) will be completed to describe distribution and severity of current symptoms. Current medication intake will also be recorded. Active range of motion of the cervical spine and upper limbs will be assessed and any pain or restriction in range noted (see table 1 for details). A neurological assessment of the upper extremities will be undertaken of cutaneous hypersensitivity and hypoaesthesia using neurotips and cotton wool, and coins will be used to assess thermal sensitivity. Isometric muscle strength and tendon reflexes will also be assessed (table 1).

Nerve mechanical sensitivity testing

Test of nerve trunk mechanical sensitivity of the brachial plexus, median and ulnar nerves will be performed bilaterally. The upper limb neurodynamic test-1 (ULNT1; median nerve bias (39), will be performed as per standard sequence (40) using 90° abduction and lateral rotation of the shoulder, elbow supination, wrist and finger extension followed by elbow extension. The upper limb neurodynamic test-3 (ULNT3; ulnar nerve bias (40) will be performed using shoulder abduction, forearm pronation, wrist and finger extension followed by elbow flexion. The ULNT1 and ULNT3 tests are considered ‘positive’ when there is reproduction of participant’s symptoms and symptoms are changed with the addition of a structural differentiation manoeuvre (39)(table 1) . For the ULNT1, elbow range of flexion at the point of symptom onset will be measured using a digital inclinometer (Trend, DLB Swansea, UK). Mechanosensitivity to pressure will be established by both response to digital pressure and an algometer (tip size = 1cm^2, Wagner, USA). The algometer will be used over the ulnar nerve at the proximal cubital tunnel and the carpal tunnel bilaterally. Other sites (brachial plexus in the supraclavicular area, median nerve at the proximal wrist crease, ulnar nerve

For peer review only - http://bmjopen.bmj.com/site/about/guidelines.xhtml

at Guyon's canal) will be palpated digitally and graded according to symptom response (see table 1). Phalen's and Tinel's test will be performed bilaterally at the wrists (41).

Quantitative sensory testing

QST will be carried out over the ventral aspect of the proximal phalanx of the index finger and the thenar eminence according to the German Network for Neuropathic Pain protocol (42). Thermal detection and pain thresholds (heat and cold) will be measured using a Thermotester (Somedic, Sweden) and mechanical detection and pain thresholds with von Frey filaments (Optihair 2 MRC systems, Germany), Rydel Seiffer tuning fork, an algometer (Wagner, USA) and weighted pin-prick stimulators (MRC Systems, Germany). QST will be carried out on the most symptomatic side in WAD2 participants and non-dominant side in healthy control participants.

MRI

Images of the brachial plexus and median nerve at the wrist (on the most symptomatic side in patients and dominant side in healthy control participants) will be obtained at each research site using a 3-Tesla scanner of the same manufacturer and model (Siemens Prisma, Siemens Medical Solutions, Erlangen, Germany) with a dedicated 64 channel head/neck coil and a small 4 channel flex coil for the wrist.

For brachial plexus imaging, participants will be positioned supine. Coronal images will be obtained using a 2D multislice T1-weighted (slice thickness = 3mm) and a T2-weighted short tau inversion recovery (STIR) 3D SPACE sequence (slice thickness = 0.8mm). For the wrist, participants will position their arm above their head in a reverse superman position with the wrist in the centre of the bore using a flex coil. Axial images will be obtained using a T1-weighted (slice thickness = 3.0mm) and a T2-weighted short tau inversion recovery (STIR) sequence with flow suppression (slice thickness = 1.5 mm). A 3D balanced steady-state free precession (bSSFP) will also be acquired as anatomical reference. The sequence will be repeated twice, with phase cycling, to minimise banding artifacts (26).

Diffusion tensor images (DTI) will also be obtained. For the brachial plexus, we will use RESOLVE sequences (43), which provide data with low distortions and clinically feasible scan times. For the wrist, we will use diffusion-weighted echo-planar imaging. This acquisition was preferred for the wrist because it provides higher signal-to-noise than RESOLVE (26). DTI images will be registered to structural images using T1 weighted images for the neck and bSSFP for the wrist.

For peer review only - http://bmjopen.bmj.com/site/about/guidelines.xhtml

In the unlikely event of a structural abnormality being noted incidentally, a radiologist or neurologist would be asked to review the scan and, if there remained any concerns, the patient would be contacted through their GP or directly by the neurologist to arrange a formal diagnostic scan.

Serum collection

Approximately 30ml of blood will be taken from each participant (BD Vacutainer Tube SST Advance). Serum will be extracted and stored at -80°C in compliance with The Human Tissue Act 2004. Levels of proteins associated with inflammation, such as pro-inflammatory cytokines, will be measured using electrochemiluminescence.

Sub study: skin biopsies

For the sub-study, two 3mm diameter skin samples will be taken from the ventrolateral aspect of the proximal phalanx of the index finger and 10cm above the contralateral lateral malleolus. We will fix the biopsies in a paraformaldehyde solution and perform immunohistochemistry to determine the integrity of dermal and intraepidermal nerve fibres as previously detailed (44, 45).

Data storage and Analysis

Data and samples will be anonymously coded, and stored on a secure web platform (REDCap, Vanderbilt University, Nashville, USA) and university freezers. Anonymised imaging data will be stored in DICOM and NIfTI formats on secure university servers. Participants will be identified by a unique study specific code.

Image analysis

T2-weighted signal intensity will be analysed using MATLAB (Mathworks Inc., Natick, USA, 2022A). Briefly, regions of interest within the brachial plexus and the median nerve will be identified, and a binary mask created to allow for background subtraction. Following background subtraction, the mean pixel grayscale value for each nerve segment will be determined and normalised to a region of adjacent tissue.

Brachial plexus DTI data will be de-noised using a self-supervised machine learning approach (46) implemented in Dipy (47). Wrist DTI data will be denoised using a method based on random matrix theory (48), implemented in MRtrix. Subsequently, they will be corrected for susceptibility and eddy

For peer review only - http://bmjopen.bmj.com/site/about/guidelines.xhtml

For peer review only

current distortions using a combination of TopUp and Eddy (part of the FMRIB's software library). DTI fitting will be performed for both kinds of data using tools from the FMRIB's software library to create fractional anisotropy (FA), mean diffusivity (MD) and other parametric maps (26). Mean DTI parameters will be determined within regions of interest.

Sample size calculation

Mean nerve signal intensity ratio data from our previous cross-sectional study of 13 controls and 9 participants with chronic WAD2 were analysed using a linear mixed model (9). The difference in means between controls and WAD2 participants was 0.0600 (standard error = 0.0315) for the C5-C8 roots of the brachial plexus, which was statistically significant; age was not a statistically significant covariate. The intraclass correlation coefficient was 0.45. Assuming that there are 8 assessments per participant (2 sides x 4 roots), a standard deviation of 0.1, intraclass correlation coefficient of 0.45, and a ratio of 3 participants with whiplash to one healthy control, the study would require 48 participants with whiplash and 16 controls to detect an effect estimate of 0.06 for the mean nerve signal intensity difference (80% power, 5% significance). Since in the acute stage it is estimated that only 50% of participants with whiplash will develop chronicity (49), we will need to image 96 participants with whiplash and 32 controls for objective 1 (difference in neuroinflammation in the acute stage). Based on ~48 participants who have not recovered at six months, we would be able to fit a logistic regression model with 5-10 predictor variables for objective 2 (predictive factors of chronicity).

For objective 3 (development of neuroinflammation over time), we will recruit 25 participants with whiplash and 25 healthy controls, which will enable an effect size of 0.9 to be detected using analysis of covariance, with 95% power for 5% significance. This value assumes that the correlation between the acute and chronic measurements is 0.5.

For objective 4 (validity of low cost tests of neuroinflammation), with 9 independent variables, a rule of thumb for a multiple linear regression (i.e. 10 participants per additional variable) implies that data from 90 participants with whiplash should be adequate to explore this (50).

For the sub-study, 68 participants with whiplash and 34 healthy controls (80% power, 5% significance, 0.53 effect size) would allow the detection of a 20% smaller difference in intraepidermal nerve fibre density in the acute stage compared to healthy controls based on our previous cohort of patients with chronic WAD2 (51).

For peer review only - http://bmjopen.bmj.com/site/about/guidelines.xhtml

Previous longitudinal studies on WAD2 have demonstrated a <15% dropout rate over six months (52, 53), and therefore we will recruit 115 participants with acute WAD2 and 34 healthy control participants.

Statistical analysis

Data will be exported to Stata (Version 17, StataCorp LLC, Texas, USA) for analysis. The distribution of the data will be checked for normality, and parametric, or non-parametric methods will be used as appropriate. Participants' characteristics will be described at all time points.

For objective 1, regression analysis, or non-parametric tests, will be used as appropriate to identify differences in MRI parameters (T2-weighted STIR 4 nerve roots per side, nerve morphology and DTI parameters) and clinical tests (e.g., neuropathic pain scores, tests of nerve mechanosensitivity, QST and serum inflammatory markers) between acute WAD2 participants and age/gender-matched controls. Regression analysis will be used to assess associations with age and height.

For objective 2, patients' recovery will be determined at 6 months with the Neck Disability Index and a global recovery question. The analysis will be conducted with three models using different predictors measured in the acute stage: a) MRI parameters of neuroinflammation, b) clinical parameters that reflect neuroinflammation and c) the addition of MRI to clinical parameters to determine whether this improves predictive strength. The dependence between MRI and clinical assessments will be investigated and composite measures added to the model. In addition, a linear regression will be performed of Neck Disability Index scores on measures of neuroinflammation.

For objective 3, mixed effects modelling will be used, and 95% confidence intervals reported, to compare MRI and clinical measures between recovered and non-recovered participants with whiplash over time.

For objective 4, multivariable regression will be used to investigate the relationship of each MRI variable with the clinical parameters of neuroinflammation. In addition, a regression analysis will be performed between clinical measures of neuroinflammation and MRI parameters.

For the sub-study, independent t-tests or nonparametric alternatives will be used to compare dermal and intraepidermal nerve fibre density in skin biopsies in patients in the acute phase after whiplash injury with healthy controls. Separate regression models will be used to investigate whether changes in nerve fibre density in the acute stage predict pain persistence at 6 months.

Data reduction techniques (e.g., principal components analysis) may be used prior to regression modelling.

For peer review only - http://bmjopen.bmj.com/site/about/guidelines.xhtml

Standardisation across the 2 sites

All clinical test procedures will be piloted between sites to ensure standardisation. Intertester reliability of range of motion using the inclinometer for nerve mechanical sensitivity testing will be performed. The German Network of Neuropathic Pain QST protocol has shown to have substantial inter-rater and inter-centre reliability (54, 55). The same MR scanners and coils are available at both sites so that settings will be identical. Any differences between sites will be adjusted at the analysis stage by comparing healthy controls at both sites.

Several quality control checks will be performed including checking of data entry (exchange of data sets between sites) and a comparison of healthy control data obtained at both sites.

Data sharing

Project data will be made available via open access publications, as well as the study webpage and the Universities of Sussex and Oxford repository, which are openly accessible. A statement will be placed on the study webpage and publications asking new users to contact the principal investigator if they wish to have access to the original data set. Data will be made accessible upon reasonable request once published or following completion of the study.

ETHICS AND DISSEMINATION

The study has received sponsorship from the University of Sussex and ethical approval from the London-Brighton & Sussex Research Ethics Committee (20/PR/0625) and South Central – Oxford C Ethics Committee (18/SC/0263).

The results of this study will be of great interest to people after whiplash injury, clinicians, researchers, legal and insurance companies and policy makers. Dissemination via a multitude of platforms is therefore necessary to enhance its reach. We plan to publish traditionally in open access peer reviewed journals as well as presenting at national and international conferences. In addition, dissemination will occur via social media outlets. Patient and clinician information resources such as infographics will be produced and disseminated. Additionally, the PPI group will support best strategies for dissemination to reach patient groups as well as policy makers. Local presentations to stakeholder groups such as patient groups, health authorities and professional bodies will be given.

For peer review only

AUTHOR CONTRIBUTIONS

AD, AS and JG conceptualised the study. CR, JF, JG, AS, AD wrote the manuscript, SB oversaw the statistical aspects of the protocol and MC determined MRI sequence and analyses. All authors reviewed drafts of the manuscript and approved the final version.

Funding statement: This work is supported by a Pain Challenge Grant from Versus Arthritis (number [22465]). ABS is supported by a Wellcome Trust Clinical Career Development Fellowship (222101/Z/20/Z) and the Medical Research Foundation (MRF-160-0013-ELP-SCHM-C0842). Her research is supported by the National Institute for Health Research (NIHR) Oxford Biomedical Research Centre (BRC). The views expressed are those of the authors and not necessarily those of the NHS, the NIHR or the Department of Health. This research was funded in whole, or in part, by the Wellcome Trust. For the purpose of Open Access, the author has applied a CC BY public copyright license to any Author Accepted Manuscript version arising from this submission.

Competing interests There were no competing interests from the authors.

For peer review only

For peer review only - http://bmjopen.bmj.com/site/about/guidelines.xhtml

REFERENCES

1. Joslin CC, Khan SN, Bannister GC. Long-term disability after neck injury. a comparative study. J Bone Joint Surg Br. 2004;86(7):1032-4.
2. Spitzer WO, Skovron ML, Salmi LR, Cassidy JD, Duranceau J, Suissa S, et al. Scientific monograph of the Quebec Task Force on Whiplash-Associated Disorders: redefining "whiplash" and its management. Spine (Phila Pa 1976). 1995;20(8 Suppl):1S-73S.
3. Tournier C, Hours M, Charnay P, Chossegros L, Tardy H. Five years after the accident, whiplash casualties still have poorer quality of life in the physical domain than other mildly injured casualties: analysis of the ESPARR cohort. BMC Public Health. 2016;16:13.
4. Commission MA. Clinical guidelines for best practice management of acute and chronic whiplash-associated disorders. 2008.
5. Walton DM, Macdermid JC, Giorgianni AA, Mascarenhas JC, West SC, Zammit CA. Risk factors for persistent problems following acute whiplash injury: update of a systematic review and meta-analysis. J Orthop Sports Phys Ther. 2013;43(2):31-43.
6. Rushton A, Wright C, Heneghan N, Eveleigh G, Calvert M, Freemantle N. Physiotherapy rehabilitation for whiplash associated disorder II: a systematic review and meta-analysis of randomised controlled trials. BMJ Open. 2011(1):e000265.
7. Teasell R, McClure J, Walton D, Pretty J, Salter K, Meyer M, et al. A research synthesis of therapeutic interventions for whiplash-associated disorder (WAD): Part 4 – noninvasive interventions for chronic WAD. Pain Research & Management. 2010;15(5):313-22.
8. Greening J, Dilley A, Lynn B. In vivo study of nerve movement and mechanosensitivity of the median nerve in whiplash and non-specific arm pain patients. Pain. 2005;115(3):248-53.
9. Greening J, Anantharaman K, Young R, Dilley A. Evidence for Increased Magnetic Resonance Imaging Signal Intensity and Morphological Changes in the Brachial Plexus and Median Nerves of Patients With Chronic Arm and Neck Pain Following Whiplash Injury. J Orthop Sports Phys Ther. 2018;48(7):523-32.
10. Chien A, Eliav E, Sterling M. Hypoaesthesia occurs with sensory hypersensitivity in chronic whiplash--further evidence of a neuropathic condition. Man Ther. 2009;14(2):138-46.
11. Chien A, Eliav E, Sterling M. Hypoesthesia occurs in acute whiplash irrespective of pain and disability levels and the presence of sensory hypersensitivity. Clin J Pain. 2008;24(9):759-66.
12. Sterling M, Jull G, Vicenzino B, Kenardy J. Sensory hypersensitivity occurs soon after whiplash injury and is associated with poor recovery. Pain. 2003;104(3):509-17.
13. Moog M, Quintner J, Hall T, Zusman M. The late whiplash syndrome: a psychophysical study. Eur J Pain. 2002;6(4):283-94.
14. Fundaun J, Kolski M, Baskozos G, Dilley A, Sterling M, Schmid AB. Nerve pathology and neuropathic pain after whiplash injury: a systematic review and meta-analysis. Pain. 2021.
15. Pulman KG, Smith M, Mengozzi M, Ghezzi P, Dilley A. The erythropoietin-derived peptide ARA290 reverses mechanical allodynia in the neuritis model. Neuroscience. 2013;233:174-83.
16. Satkeviciute I, Dilley A. [EXPRESS] Neuritis and vinblastine-induced axonal transport disruption lead to signs of altered dorsal horn excitability. Mol Pain. 2018:1744806918799581.
17. Bove GM, Ransil BJ, Lin HC, Leem JG. Inflammation induces ectopic mechanical sensitivity in axons of nociceptors innervating deep tissues. JNeurophysiol. 2003;90(3):1949-55.
18. Dilley A, Lynn B, Pang SJ. Pressure and stretch mechanosensitivity of peripheral nerve fibres following local inflammation of the nerve trunk. Pain. 2005;117(3):462-72.
19. Goodwin G, Bove GM, Dayment B, Dilley A. Characterizing the Mechanical Properties of Ectopic Axonal Receptive Fields in Inflamed Nerves and Following Axonal Transport Disruption. Neuroscience. 2020;429:10-22.
20. Dilley A, Bove GM. Resolution of inflammation induced axonal mechanical sensitivity and conduction slowing in C-fiber nociceptors. Journal of Pain. 2008;9(2):185-92.
21. Filler AG, Howe FA, Hayes CE, Kliot M, Winn HR, Bell, et al. Magnetic resonance neurography. Lancet. 1993;341(8846):659-61.

For peer review only - http://bmjopen.bmj.com/site/about/guidelines.xhtml

22. Schwarz D, Kele H, Kronlage M, Godel T, Hilgenfeld T, Bendszus M, et al. Diagnostic Value of Magnetic Resonance Neurography in Cervical Radiculopathy: Plexus Patterns and Peripheral Nerve Lesions. Invest Radiol. 2018;53(3):158-66.
23. Baumer P, Dombert T, Staub F, Kaestel T, Bartsch AJ, Heiland S, et al. Ulnar Neuropathy at the Elbow: MR Neurography--Nerve T2 Signal Increase and Caliber. Radiology. 2011;260(1):199-206.
24. Boretius S, Gadjanski I, Demmer I, Bahr M, Diem R, Michaelis T, et al. MRI of optic neuritis in a rat model. Neuroimage. 2008;41(2):323-34.
25. Stanisz GJ, Webb S, Munro CA, Pun T, Midha R. MR properties of excised neural tissue following experimentally induced inflammation. Magnetic Resonance in Medicine. 2004;51(3):473-9.
26. Schmid AB, Campbell J, Hurley SA, Jbabdi S, Andersson JL, Jenkinson M, et al. Feasibility of Diffusion Tensor and Morphologic Imaging of Peripheral Nerves at Ultra-High Field Strength. Invest Radiol. 2018;53(12):705-13.
27. Guggenberger R, Eppenberger P, Markovic D, Nanz D, Chhabra A, Pruessmann KP, et al. MR neurography of the median nerve at 3.0T: optimization of diffusion tensor imaging and fiber tractography. Eur J Radiol. 2012;81(7):e775-82.
28. Heckel A, Weiler M, Xia A, Ruetters M, Pham M, Bendszus M, et al. Peripheral Nerve Diffusion Tensor Imaging: Assessment of Axon and Myelin Sheath Integrity. PLoS One. 2015;10(6):e0130833.
29. Song SK, Sun SW, Ju WK, Lin SJ, Cross AH, Neufeld AH. Diffusion tensor imaging detects and differentiates axon and myelin degeneration in mouse optic nerve after retinal ischemia. Neuroimage. 2003;20(3):1714-22.
30. Lin M, He H, Schifitto G, Zhong J. Simulation of changes in diffusion related to different pathologies at cellular level after traumatic brain injury. Magn Reson Med. 2016;76(1):290-300.
31. Vernon H, Mior S. The Neck Disability Index: a study of reliability and validity. J Manipulative Physiol Ther. 1991;14(7):409-15.
32. Vernon H. The Neck Disability Index: state-of-the-art, 1991-2008. J Manipulative Physiol Ther. 2008;31(7):491-502.
33. Freynhagen R, Baron R, Gockel U, Tolle TR. painDETECT: a new screening questionnaire to identify neuropathic components in patients with back pain. Curr Med Res Opin. 2006;22(10):1911-20.
34. Hansen M, Andersen TE, Armour C, Elklit A, Palic S, Mackrill T. PTSD-8: A Short PTSD Inventory. Clin Pract Epidemiol Ment Health. 2010;6:101-8.
35. Osman A, Barrios FX, Kopper BA, Hauptmann W, Jones J, O'Neill E. Factor structure, reliability, and validity of the Pain Catastrophizing Scale. J Behav Med. 1997;20(6):589-605.
36. Lovibond PF, Lovibond SH. The structure of negative emotional states: comparison of the Depression Anxiety Stress Scales (DASS) with the Beck Depression and Anxiety Inventories. Behav Res Ther. 1995;33(3):335-43.
37. Herdman M, Gudex C, Lloyd A, Janssen M, Kind P, Parkin D, et al. Development and preliminary testing of the new five-level version of EQ-5D (EQ-5D-5L). Qual Life Res. 2011;20(10):1727-36.
38. Creamer M, Bell R, Failla S. Psychometric properties of the Impact of Event Scale - Revised. Behav Res Ther. 2003;41(12):1489-96.
39. Nee RJ, Jull GA, Vicenzino B, Coppieters MW. The validity of upper-limb neurodynamic tests for detecting peripheral neuropathic pain. J Orthop Sports Phys Ther. 2012;42(5):413-24.
40. Butler D. The Sensitive nervous system: Noigroup Publications 2000.
41. Kuschner SH, Ebramzadeh E, Johnson D, Brien WW, Sherman R. Tinel's sign and Phalen's test in carpal tunnel syndrome. Orthopedics. 1992;15(11):1297-302.
42. Rolke R, Baron R, Maier C, Tölle TR, Treede DR, Beyer A, et al. Quantitative sensory testing in the German Research Network on Neuropathic Pain (DFNS): standardized protocol and reference values. Pain. 2006;123(3):231-43.

For peer review only - http://bmjopen.bmj.com/site/about/guidelines.xhtml

43. Porter DA, Heidemann RM. High resolution diffusion-weighted imaging using readout-segmented echo-planar imaging, parallel imaging and a two-dimensional navigator-based reacquisition. Magn Reson Med. 2009;62(2):468-75.
44. Baskozos G, Sandy-Hindmarch O, Clark AJ, Windsor K, Karlsson P, Weir GA, et al. Molecular and cellular correlates of human nerve regeneration: ADCYAP1/PACAP enhance nerve outgrowth. Brain. 2020;143(7):2009-26.
45. Schmid AB, Bland JD, Bhat MA, Bennett DL. The relationship of nerve fibre pathology to sensory function in entrapment neuropathy. Brain. 2014;137(Pt 12):3186-99.
46. Fadnavis S, Batson J, Garyfallidis E. Patch2Self: Denoising Diffusion MRI with Self-Supervised Learning. Proceedings of the 34th International Conference on Neural Information Processing Systems; Vancouver, Canada2020. p. 16293–303.
47. Garyfallidis E, Brett M, Amirbekian B, Rokem A, van der Walt S, Descoteaux M, et al. Dipy, a library for the analysis of diffusion MRI data. Front Neuroinform. 2014;8:8.
48. Veraart J, Fieremans E, Novikov DS. Diffusion MRI noise mapping using random matrix theory. Magn Reson Med. 2016;76(5):1582-93.
49. Carroll LJ, Holm LW, Hogg-Johnson S, Cote P, Cassidy JD, Haldeman S, et al. Course and prognostic factors for neck pain in whiplash-associated disorders (WAD): results of the Bone and Joint Decade 2000-2010 Task Force on Neck Pain and Its Associated Disorders. Spine (Phila Pa 1976). 2008;33(4 Suppl):S83-92.
50. VanVoorhis CRW, Morgan BL. Understanding Power and Rules of Thumb for Determining Sample Sizes. Tutorials in Quantitative Methods for Psychology. 2007;3(2):43-50.
51. Farrell SF, Sterling M, Irving-Rodgers H, Schmid AB. Small fibre pathology in chronic whiplash-associated disorder: A cross-sectional study. Eur J Pain. 2020;24(6):1045-57.
52. Sterling M, Elliott JM, Cabot PJ. The course of serum inflammatory biomarkers following whiplash injury and their relationship to sensory and muscle measures: a longitudinal cohort study. PLoS One. 2013;8(10):e77903.
53. Sterling M, Jull G, Vicenzino B, Kenardy J, Darnell R. Physical and psychological factors predict outcome following whiplash injury. Pain. 2005;114(1-2):141-8.
54. Middlebrook N, Heneghan NR, Evans DW, Rushton A, Falla D. Reliability of temporal summation, thermal and pressure pain thresholds in a healthy cohort and musculoskeletal trauma population. PloS one. 2020;15(5):e0233521-e.
55. Geber C, Klein T, Azad S, Birklein F, Gierthmühlen J, Huge V, et al. Test-retest and interobserver reliability of quantitative sensory testing according to the protocol of the German Research Network on Neuropathic Pain (DFNS): a multi-centre study. Pain. 2011;152(3):548-56.

For peer review only

For peer review only - http://bmjopen.bmj.com/site/about/guidelines.xhtml

For peer review only

For peer review only - http://bmjopen.bmj.com/site/about/guidelines.xhtml

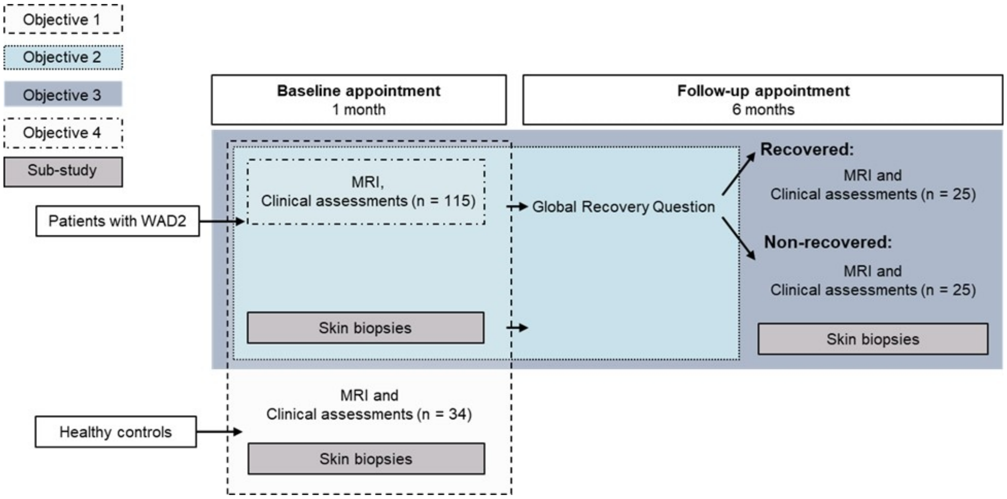

164x80mm (150 x 150 DPI)

BMJ Open

Does peripheral neuroinflammation predict chronicity following whiplash injury? Protocol for a prospective cohort study

Journal:	*BMJ Open*
Manuscript ID	bmjopen-2022-066021.R1
Article Type:	Protocol
Date Submitted by the Author:	02-Nov-2022
Complete List of Authors:	Ridehalgh, Colette; University of Brighton, School of Sport and Health Sciences; Brighton and Sussex Medical School, Neuroscience<br>Fundaun, Joel; University of Oxford, Nuffied Department of Clinical Neurosciences<br>Bremner, Stephen; Brighton and Sussex Medical School, Department of Primary Care & Public Health<br>Cercignani, Mara; Cardiff University, Cardiff University Brain Research Imaging Centre<br>Young, Rupert; University of Sussex School of Engineering and Informatics<br>Trivedy, Chetan; Brighton and Sussex University Hospitals NHS Trust; Queen Mary University of London<br>Novak, Alex; Oxford University Hospitals NHS Foundation Trust, Emergency Medicine Research Oxford<br>Greening, Jane; Brighton and Sussex Medical School, Neuroscience<br>Schmid, Annina ; University of Oxford, Nuffield Department of Clinical Neurosciences<br>Dilley, A.; Brighton and Sussex Medical School, Neuroscience
**Primary Subject Heading**:	Emergency medicine
Secondary Subject Heading:	Neurology, Radiology and imaging
Keywords:	Neuroradiology < NEUROLOGY, TRAUMA MANAGEMENT, NEUROPHYSIOLOGY, ACCIDENT & EMERGENCY MEDICINE, Neurological pain < NEUROLOGY

SCHOLARONE™
Manuscripts

Does peripheral neuroinflammation predict chronicity following whiplash injury? Protocol for a prospective cohort study

Colette Ridehalgh, Joel Fundaun, Stephen Bremner, Mara Cercignani, Rupert Young, Chet Trivedy, Alex Novak, Jane Greening, Annina Schmid, Andrew Dilley

Colette Ridehalgh (joint first author), School of Sport and Health Sciences, University of Brighton, 49 Darley Rd, Eastbourne, BN20 7UR, United Kingdom; Department of Neuroscience, Brighton and Sussex Medical School, Trafford Centre, University of Sussex, Falmer, Brighton, BN1 9RY, United Kingdom

Joel Fundaun (joint first author), Nuffield Department of Clinical Neurosciences, University of Oxford, Level 6, West Wing, John Radcliffe Hospital, Oxford, OX3 9DU, United Kingdom

Stephen Bremner, Department of Primary Care & Public Health, Brighton and Sussex Medical School, Watson Building, University of Brighton Falmer Campus, Brighton, BN1 9PH, United Kingdom

Mara Cercignani, Cardiff University Brain Research Imaging Centre, Cardiff University, Cardiff, CF24 4HQ, United Kingdom

Rupert Young, School of Engineering and Informatics, University of Sussex, Sussex House, Falmer Brighton, BN1 9RH, United Kingdom

Chet Trivedy, Emergency Department, University Hospitals Sussex NHS Foundation Trust, Princess Royal Hospital, Lewes Road, Haywards Heath, RH16 4EX, United Kingdom

Queen Mary University of London, Mile End Road, London, E1 4NS, United Kingdom

Alex Novak, Emergency Medicine Research Oxford (EMROx), Oxford University Hospitals NHS Foundation Trust, Oxford, OX3 9DU, United Kingdom

Jane Greening, Department of Neuroscience, Brighton and Sussex Medical School, Trafford Centre, University of Sussex, Falmer, Brighton, BN1 9RY, United Kingdom

Annina Schmid (joint last author), Nuffield Department of Clinical Neurosciences, University of Oxford, Level 6, West Wing, John Radcliffe Hospital, Oxford, OX3 9DU, United Kingdom

Andrew Dilley (joint last author), Department of Neuroscience, Brighton and Sussex Medical School, Trafford Centre, University of Sussex, Falmer, Brighton, BN1 9RY, United Kingdom

For peer review only - http://bmjopen.bmj.com/site/about/guidelines.xhtml

Correspondence to:

Colette Ridehalgh, School of Sport and Health Sciences, University of Brighton, 49 Darley Rd, Eastbourne, BN20 7UR, United Kingdom

cr19@brighton.ac.uk

Keywords: Whiplash injuries; neuritis; magnetic resonance imaging; peripheral nerve injuries

ABSTRACT

Introduction

Whiplash associated disorder grade 2 (WAD2) is characterised by musculoskeletal pain/ tenderness but no apparent nerve injury. However, studies have found clinical features indicative of neuropathy and neuropathic pain. This may indicate peripheral nerve inflammation, since preclinical neuritis models found mechanical sensitivity in inflamed, intact nociceptors. The primary aim of this study is to establish the contribution of peripheral neuroinflammation to WAD2 and its role in prognosis. Participants will be invited to participate in a sub-study investigating the contribution of cutaneous small fibre pathology to WAD2.

Methods and analysis

115 participants within one month following whiplash injury and 34 healthy control participants will be recruited and complete validated questionnaires for pain, function, and psychological factors. Data collection will take place at the Universities of Sussex and Oxford, UK. Clinical examination, quantitative sensory testing and blood samples will be undertaken. MRI scans using T2-weighted and diffusion tensor images of brachial plexus and wrist will determine nerve inflammation and nerve structural changes. Skin biopsies from the sub-study will determine structural integrity of dermal and intraepidermal nerve fibres. At six months, we will evaluate recovery using Neck Disability Index and a self-rated global recovery question and repeat the outcome measures. Regression analysis will identify differences in MRI parameters, clinical tests and skin biopsies between WAD2 participants and age/gender-matched controls. Linear and logistic regression analyses will assess if nerve inflammation (MRI parameters) predicts poor outcome. Mixed effects modelling will compare MRI and clinical measures between recovered and non-recovered participants over time.

Ethics and dissemination

Ethical approval was received from London-Brighton & Sussex Research Ethics Committee (20/PR/0625) and South Central–Oxford C Ethics Committee (18/SC/0263). Written informed

For peer review only - http://bmjopen.bmj.com/site/about/guidelines.xhtml

consent will be obtained from participants prior to participation in the study. Results will be disseminated through publications in peer-reviewed journals, presentations at national/international conferences and social media.

ARTICLE SUMMARY

Strengths and limitations of this study

- This study will evaluate the contribution of peripheral nerve inflammation to whiplash associated disorder grade 2 and its role in prognosis.
- The design will enable us to understand if simple clinical tests can be used to identify nerve inflammation.
- One potential limitation of a two-site study can be variation in data collection between sites, although regular meetings and site visits have been put in place to minimise this.
- The use of different scanners could result in non-homogenous data sets; however, identical scanner types are being used, which should reduce the impact of this potential issue.
- It is not possible to blind the examiner to the status of patients versus healthy control participants, which may influence digital nerve palpation; therefore, care will be taken to ensure similar pressures are applied to both groups where possible.

INTRODUCTION

Whiplash injury sustained by a motor vehicle collision can cause significant long-term pain and disability. In the UK, economic costs are as high as £3 billion per year mostly due to healthcare costs (1). Most whiplash cases are considered whiplash associated disorder grade 2 (WAD2), as defined by the Quebec Task Force Classification system (2). WAD2 refers to musculoskeletal pain and tenderness but with no frank nerve injury identified during routine clinical testing (e.g., electrodiagnostic testing, neurological bedside testing). Approximately 50% of people with WAD2 develop chronic symptoms that frequently persist for more than five years (3, 4). Although a limited number of prognostic factors have been identified (5), they do not indicate a site of tissue pathology. This lack of understanding of the underlying pathophysiology likely explains why treatments for this condition are relatively ineffective (6, 7). Despite the absence of a detectable nerve injury on routine clinical testing, many of the clinical features of WAD2 indicate a syndrome that is neuropathic in nature, such as reduced vibration thresholds, cutaneous thermal hypoaesthesia and cutaneous hypersensitivities to tactile and thermal stimuli distal to the site of injury (3, 8-14). Furthermore, clinical tests of nerve trunk mechanical sensitivity, such as palpating over upper limb peripheral

For peer review only

nerve trunks and positioning the upper limb in postures that increases the tension in the brachial plexus and median nerve (the upper limb neurodynamic test-1; ULNT1), provoke symptoms in many patients (8, 9, 14). Based on these findings, we propose that some people diagnosed with WAD2 may have an undetected nerve pathology, such as peripheral neuroinflammation, which could be a cause of chronicity. Consistent with a role for neuroinflammation, animals with a localised neuritis develop tactile and thermal-evoked cutaneous hypersensitivities in the absence of axonal degeneration (15-17). Of note, intact nociceptive axons become mechanically sensitive responding to pressure and stretch (17-20), similar to the neural mechanosensitivity observed in people with WAD2.

Magnetic resonance (MR) neurography has gained increasing interest in the diagnosis of peripheral nerve pathology (21-23). An increased signal intensity on T2-weighted sequences is considered an imaging correlate of the intraneural oedema associated with peripheral neuroinflammation. Accordingly, T2-weighted signal is increased in animal models of neuroinflammation (24, 25), and correlates positively with histological findings of inflammation (25). Using T2-weighted MR neurography in a group of patients with chronic WAD2, we have identified increased signal intensity in the cervical nerve roots and the median nerve at the carpal tunnel, indicative of peripheral neuroinflammation (9). In addition to T2-weighted MR neurography, diffusion tensor imaging (DTI) has recently been employed to examine the microstructural integrity of peripheral nerves (26, 27). DTI evaluates the diffusivity of water molecules within nerves, which can provide information on the integrity of axons and myelin sheaths (28, 29), as well as intraneural oedema (30).

In addition to neuroinflammation and functional changes to the nervous system, there is also some evidence for the presence of structural nerve pathology in patients with WAD2. This is apparent by a decreased intraepidermal nerve fibre density in the index finger in patients with chronic WAD2 (14). It currently remains unknown whether such small fibre degeneration is already apparent in the early stages after injury and whether it predicts outcome.

The main aim of this study is to establish the contribution of peripheral nerve pathology to WAD2 and its role in prognosis. Our primary focus is on peripheral neuroinflammation, which we will evaluate using a detailed examination, including MRI of the brachial plexus and distal nerve trunks, blood markers and clinical tests of nerve trunk mechanical sensitivity. Additionally, recruited participants will be invited to participate in a sub-study that will use skin biopsies to establish the contribution of cutaneous nerve fibre pathology to WAD2. Our results will advance our understanding of the pathology underlying WAD2 and may identify factors that predict the development of persistent symptoms.

For peer review only - http://bmjopen.bmj.com/site/about/guidelines.xhtml

METHODS AND ANALYSIS

Objectives

The first objective of this study is to determine the presence of peripheral neuroinflammation during the acute stage following a WAD2 injury. This objective will use MRI to assess the extent of peripheral neuroinflammation and nerve microstructure in a cross-section of patients within 4 weeks of injury and healthy controls. The second objective will determine whether the presence of peripheral neuroinflammation in the acute stage can predict patient recovery status at six months. The third objective will determine the course of peripheral neuroinflammation from the acute to chronic stages of a WAD2 injury. For this objective, we will examine changes in MRI measures and clinical tests from baseline to six months in both recovered and non-recovered patients. The fourth objective will determine whether low-cost clinical tests can serve as markers to detect neuroinflammation as determined on MRI. The objective of the sub-study is to determine whether the structural integrity of cutaneous nerve fibres is altered in the acute stage following a WAD2 injury, and whether it contributes to the progression to chronicity.

Study design

This is a prospective cohort study at two centres. A total of 115 participants will be recruited following a whiplash injury, within one month of their original injury. We will also recruit 34 healthy proportionally age- and gender-matched control participants. People with acute WAD2 and healthy control participants will attend a baseline appointment. In addition to MRI, a detailed musculoskeletal assessment will be performed, as well as tests of nerve trunk mechanical sensitivity and quantitative sensory testing (QST). A blood sample will be taken and analysed for levels of inflammatory markers. Participants will also be asked to complete a series of validated questionnaires to assess neuropathic symptoms, functional deficits, quality of life and psychological factors. For the sub-study, skin biopsies from the index finger and ankle will also be taken. At six months, the Neck Disability Index and a self-rated global recovery question will be used to determine recovery. A sequential sample of patients will be invited to attend a follow-up appointment during which the same detailed assessments will be repeated (Figure 1).

This study has been registered at ClinicalTrials.gov reference number NCT04940923. The protocol version is V2 25/9/20.

For peer review only - http://bmjopen.bmj.com/site/about/guidelines.xhtml

Patient and public involvement

Patient partners were involved in the design of the study and highlighted the relevance and need for this study. They will continue to be an integral part of the research team, offering advice from conducting the study through to interpretation of the data and dissemination of findings.

Participant recruitment

Patients aged 18-60 that meet the Quebec Task Force Classification of WAD2 will be recruited within four weeks of injury. Participants will be recruited from emergency departments and minor injury units in Surrey, Sussex, Kent and Oxfordshire directly by clinicians, and following weekly hospital database searches using defined search terms (e.g., motor vehicle collision, muscle injury: neck, bruise / contusion / abrasion: neck, sprain / ligament injury: cervical spine). Potential participants will be contacted in person, or by phone, and asked for permission for their details to be passed on to the study team, and/or sent the study details. In addition, posters will be displayed in departments with study contact details. Thirty-four healthy age- and gender-matched control participants will be recruited through departmental emailing lists (Universities of Sussex and Oxford as well as local hospitals), social media and flyers on public notice boards. Potential participants will be screened for suitability via telephone and also be invited to take part in the sub-study. Eligible individuals will be sent a participant information sheet and questionnaires to complete (see below) and will be invited to attend a baseline appointment at either Brighton and Sussex Medical School, University of Sussex, Falmer, or the Nuffield Department of Clinical Neurosciences, University of Oxford.

Exclusion criteria include pregnancy, recent history of cervical/arm pain lasting >3 months, previous diagnosis of a peripheral neuropathy, history of systemic illness or autoimmune disease, non-medically controlled hypertension, ongoing steroid treatment. Participants will be assessed for their suitability to undergo MRI. Additionally, healthy control participants must not have had a history of whiplash injury and have not received treatment for neck, thoracic spine or upper limb pain within the past 3 months.

Both recruitment methods and experimental design, in particular length of time of research appointments and MRI scanning, have been discussed and modified based on the comments received from our patient advisory group.

For peer review only - http://bmjopen.bmj.com/site/about/guidelines.xhtml

Baseline and follow-up appointment

Written informed consent will be obtained by a member of the study team at the baseline appointment. Additionally, consent will be gained for use of data/samples in ancillary studies. The following procedures will be carried out (see table 1 for full details):

Table 1. Summary of self-reported and physical assessments

Test	Description	Type of tool/assessment	Participants
**Demographics**	Age, gender, height/weight	Self-reported	HC and whiplash
**Injury details**	Impact, anticipation	Self-reported	Whiplash
**Medication details**	Specific to WAD symptoms	Self-reported	Whiplash
**Symptom history**	Description of pain or symptoms	Self-reported	Whiplash
**Pain level**	Current pain severity	Visual analogue scale	Whiplash
**Management**	Any investigations/treatment	Self-reported	Whiplash
**Type of pain**	Neuropathic Pain	pain DETECT Questionnaire	Whiplash
**Disability**	Neck disability	Neck Disability Index	Whiplash
**Psychological factors**	Posttraumatic stress<br>Pain catastrophising<br><br>Depression, anxiety and stress<br>Quality of life	PTSD-8<br>Pain Catastrophising Scale<br>DASS 42<br><br>EQ-5D-5L	Whiplash
**Active range of motion**	Cervical spine<br>Glenohumeral joints<br>Elbow, wrist and hands	0-3 scale (0 =full range no symptoms, 1 full range, symptoms, 2 reduced range, no symptoms, 3 reduced range, symptoms)	HC and whiplash
**Standardised neurological examination**	Sensation (dermatomes C5-T1)<br>Myotomes (C5-T1)<br><br>Reflexes (biceps, triceps)	Cotton wool (light touch)<br>Neurotip (pin prick)<br>Isometric muscle contraction<br>Reflex hammer	HC and whiplash
**Bedside sensory testing**	Sensation- main pain area and index finger (median nerve/C6 innervation)	Warm/cold coin (thermal) and Neurotip (pinprick)	HC and whiplash
**Quantitative sensory testing- index finger**	Warm/cold sensation/pain thresholds<br>Mechanical detection thresholds	MSA thermal stimulator (Somedic, Stockholm, Sweden)<br>Von Frey filaments (Marstock NerveTest filaments	HC and whiplash

For peer review only - http://bmjopen.bmj.com/site/about/guidelines.xhtml

	Mechanical pain thresholds	(MRCsystems, Heidelberg, Germany<br>Weighted pin prick stimulators (MRC systems, Heidelberg, Germany)<br>Vibration (Tuning fork)<br>Pressure pain threshold (Algometer, Wagner Force dial, Greenwich, USA)	
**Heightened nerve mechanosensitivity**	Upper limb neurodynamic test 1 (median nerve bias)<br><br>Upper limb neurodynamic test 3 (ulnar nerve bias)<br><br>Nerve palpation<br>Brachial plexus<br>Median nerve proximal to carpal tunnel<br>Ulnar nerve (Guyon's canal)<br><br>Ulnar nerve (proximal to cubital tunnel)<br>Median nerve (carpal tunnel)<br><br>Phalens<br>Tinels	Positive/negative test (symptom response changes with structural differentiation)<br>Inclinometer (ROM degrees)<br><br>Positive/negative test (symptom response changes with structural differentiation)<br><br>Digital palpation (0-3: 0 = No pain or discomfort<br>1= local discomfort<br>2a= local painful response<br>2b=referred pain/symptoms<br><br>Algometer and score (0-3)<br><br>Digital palpation (0-1)	HC and whiplash
**Magnetic Resonance Imaging**	Brachial plexus<br>Wrist	T2 weighted images<br>Diffusion tensor images	HC and whiplash
**Serum samples**	venepuncture	inflammatory markers	HC and whiplash
**Skin samples**	Skin biopsy	Density of dermal and intraepidermal nerve fibres using immunohistochemistry	HC and whiplash

For peer review only - http://bmjopen.bmj.com/site/about/guidelines.xhtml

Key: WAD whiplash associated disorder; HC healthy controls; ROM range of motion; PTSD posttraumatic stress disorder; DASS depression, anxiety and stress sub-scale

Questionnaires

Patients will complete the following validated questionnaires: Neck Disability Index (NDI) (31, 32), pain detect questionnaire (painDETECT) (33), short post-traumatic stress inventory (PTSD8) (34), pain catastrophising scale (PCS) (35), depression, anxiety and stress scale (DASS42) (36), the quality of life questionnaire (EQ-5D-5L) (37), and the Impact of Events Scale (IES-Revised) (38).

Clinical assessment

Demographic information and type of neck injury sustained will be obtained (i.e., speed of impact, direction of collision, and initial symptoms). A body chart and visual analogue scale (0-100 mm) will be completed to describe distribution and severity of current symptoms. Current medication intake will also be recorded. Active range of motion of the cervical spine and upper limbs will be assessed and any pain or restriction in range noted (see table 1 for details). A neurological assessment of the upper extremities will be undertaken of cutaneous hypersensitivity and hypoaesthesia using neurotips and cotton wool, and coins will be used to assess thermal sensitivity. Isometric muscle strength and tendon reflexes will also be assessed (table 1).

Nerve mechanical sensitivity testing

Test of nerve trunk mechanical sensitivity of the brachial plexus, median and ulnar nerves will be performed bilaterally. The upper limb neurodynamic test-1 (ULNT1; median nerve bias (39), will be performed as per standard sequence (40) using 90° abduction and lateral rotation of the shoulder, elbow supination, wrist and finger extension followed by elbow extension. The upper limb neurodynamic test-3 (ULNT3; ulnar nerve bias (40) will be performed using shoulder abduction, forearm pronation, wrist and finger extension followed by elbow flexion. The ULNT1 and ULNT3 tests are considered 'positive' when there is reproduction of participant's symptoms and these are changed with the addition of a structural differentiation manoeuvre (39)(table 1) . For the ULNT1, elbow range of flexion at the point of symptom onset will be measured using a digital inclinometer (Trend, DLB Swansea, UK). Mechanosensitivity to pressure will be established by both response to digital pressure and using an algometer (tip size = 1cm^2, Wagner, USA). The algometer will be used over the ulnar nerve at the proximal cubital tunnel and the carpal tunnel bilaterally. Participants will be asked to inform the researcher at the point where the pressure applied over the nerve changes to

For peer review only - http://bmjopen.bmj.com/site/about/guidelines.xhtml

For peer review only

pain. Measurements will be taken three times at each site and averages used for analysis. Other sites (bilaterally at brachial plexus in the supraclavicular area, median nerve at the proximal wrist crease, ulnar nerve at Guyon's canal) will be palpated digitally and graded according to symptom response (see table 1). Phalen's and Tinel's test will be performed bilaterally at the wrists (41).

Quantitative sensory testing

QST will be carried out over the ventral aspect of the proximal phalanx of the index finger and the thenar eminence according to the German Network for Neuropathic Pain protocol (42). Additionally, we will perform warm detection, cold detection and pressure pain thresholds over the contralateral lower limb (upper anterolateral aspect of the tibia) to assess for any systemic changes to QST. Thermal detection and pain thresholds (heat and cold) will be measured using a Thermotester (Somedic, Sweden) and mechanical detection and pain thresholds with von Frey filaments (Optihair 2 MRC systems, Germany), Rydel Seiffer tuning fork, an algometer (Wagner, USA) and weighted pin-prick stimulators (MRC Systems, Germany). QST will be carried out on the most symptomatic side in WAD2 participants and non-dominant side in healthy control participants.

Magnetic Resonance Imaging (MRI)

Images of the brachial plexus and median nerve at the wrist (on the most symptomatic side in patients and dominant side in healthy control participants) will be obtained at each research site using a 3-Tesla scanner of the same manufacturer and model (Siemens Prisma, Siemens Medical Solutions, Erlangen, Germany) with a dedicated 64 channel head/neck coil and a small 4 channel flex coil for the wrist.

For brachial plexus imaging, participants will be positioned supine. Coronal images will be obtained using a 2D multislice T1-weighted (slice thickness = 3mm) and a T2-weighted short tau inversion recovery (STIR) 3D SPACE sequence (slice thickness = 0.8mm). For the wrist, participants will position their arm above their head in a reverse superman position with the wrist in the centre of the bore using a flex coil. Axial images will be obtained using a T1-weighted (slice thickness = 3.0mm) and a T2-weighted short tau inversion recovery (STIR) sequence with flow suppression (slice thickness = 1.5 mm). A 3D balanced steady-state free precession (bSSFP) will also be acquired as anatomical reference. The sequence will be repeated twice, with phase cycling, to minimise banding artifacts (26).

For peer review only - http://bmjopen.bmj.com/site/about/guidelines.xhtml

Diffusion tensor images (DTI) will also be obtained. For the brachial plexus, we will use RESOLVE sequences (43), which provide data with low distortions and clinically feasible scan times. For the wrist, we will use diffusion-weighted echo-planar imaging. This acquisition was preferred for the wrist because it provides higher signal-to-noise than RESOLVE (26). DTI images will be registered to structural images using T1 weighted images for the neck and bSSFP for the wrist.

In the unlikely event of a structural abnormality being noted incidentally, a radiologist or neurologist would be asked to review the scan and, if there remained any concerns, the patient would be contacted through their GP or directly by the neurologist to arrange a formal diagnostic scan.

Serum collection

Approximately 30ml of blood will be taken from each participant (BD Vacutainer Tube SST Advance). Serum will be extracted and stored at -80°C in compliance with The Human Tissue Act 2004. Levels of proteins associated with inflammation, such as pro-inflammatory cytokines, will be measured using electrochemiluminescence. All serum samples will be collected and stored in accordance with the Human Tissue Act.

Sub study: skin biopsies

For the sub-study, two 3mm diameter skin samples will be taken from the ventrolateral aspect of the proximal phalanx of the index finger on the more affected side and 10cm above the contralateral lateral malleolus under sterile conditions using local anaesthesia (1% lidocaine, 1 – 2.0 mL). We will fix the biopsies in a periodate-lysine-paraformaldehyde solution for 30 minutes followed by washing samples in 0.1 M phosphate buffer. The tissue will be embedded in OCT compound and stored at -80° Celsius. Fifty µm sections will be cut and immunohistochemistry will be performed to visualise cutaneous nerve fibres (e.g. with protein gene product 9.5). A single, blinded assessor will evaluate the integrity of intraepidermal and dermal nerve fibres, as previously detailed (44-46). In brief, intraepidermal nerve fibre density will be counted through the microscope from three samples per patient with the average expressed as fibres per mm epidermis(46). Dermal innervation will be established by counting the number of dermal nerve bundles per mm^2 dermis that include at least five axons (47). All skin samples will be collected and stored in accordance with the human tissue act.

For peer review only - http://bmjopen.bmj.com/site/about/guidelines.xhtml

For peer review only

Follow-up

All participants will be asked to complete the questionnaires 6 months after their injury. Twenty five participants who have recovered and 25 who have not recovered will undergo the full protocol. To promote participant retention, a newsletter will be sent to participants to keep them up to date about the study, and a small payment will be given to those who return their completed questionnaires at 6 months or attend their follow-up appointment.

Data storage and analysis

Data and samples will be anonymously coded, and stored on a secure web platform (REDCap, Vanderbilt University, Nashville, USA) and university freezers respectively. Anonymised imaging data will be stored in DICOM and NIfTI formats on secure university servers. Participants will be identified by a unique study specific code. All data and sample analyses will be blinded to participant group and timepoint.

Image analysis

MRI data will be coded and analysed blind. T2-weighted signal intensity will be analysed using MATLAB (Mathworks Inc., Natick, USA, 2022A). Briefly, regions of interest within the brachial plexus and the median nerve will be identified, and a binary mask created to allow for background subtraction. Following background subtraction, the mean pixel grayscale value for each nerve segment will be determined and normalised to a region of adjacent tissue.

Brachial plexus DTI data will be de-noised using a self-supervised machine learning approach (48) implemented in Dipy (49). Wrist DTI data will be denoised using a method based on random matrix theory (50), implemented in MRtrix. Subsequently, they will be corrected for susceptibility and eddy current distortions using a combination of TopUp and Eddy (part of the FMRIB's software library). DTI fitting will be performed for both kinds of data using tools from the FMRIB's software library to create fractional anisotropy (FA), mean diffusivity (MD) and other parametric maps (26). Mean DTI parameters will be determined within regions of interest.

Sample size calculation

Mean nerve signal intensity ratio data from our previous cross-sectional study of 13 controls and 9 participants with chronic WAD2 were analysed using a linear mixed model (9). The observed difference in means between controls and WAD2 participants was 0.0600 (standard error = 0.0315)

For peer review only - http://bmjopen.bmj.com/site/about/guidelines.xhtml

for the C5-C8 roots of the brachial plexus, which was statistically significant; age was not a statistically significant covariate. The intraclass correlation coefficient of the nerve signal ratio was estimated to be 0.45. Assuming that there are 8 assessments per participant (2 sides x 4 roots), a standard deviation of 0.1, intraclass correlation coefficient of 0.45, and a ratio of 3 participants with whiplash to one healthy control, the study would require 48 participants with whiplash and 16 controls to detect an effect estimate of 0.06 for the mean nerve signal intensity difference (80% power, 5% significance). Since in the acute stage it is estimated that only 50% of participants with whiplash will develop chronicity (51), we will need to image 96 participants with whiplash and 32 controls for objective 1 (difference in neuroinflammation in the acute stage). Based on ~48 participants who have not recovered at six months, we would be able to fit a logistic regression model with 5-10 predictor variables for objective 2 (predictive factors of chronicity).

For objective 3 (development of neuroinflammation over time), we will ask 25 participants who have recovered and 25 participants who have not recovered to return at 6 months, which will enable an effect size of 0.9 to be detected using analysis of covariance, with 95% power for 5% significance. This value assumes that the correlation between the acute and chronic measurements is 0.5.

For objective 4 (validity of low cost tests of neuroinflammation), with 9 independent variables, a rule of thumb for a multiple linear regression (i.e. 10 participants per additional variable) implies that data from 90 participants with whiplash should be adequate to explore this (52).

For the sub-study, 68 participants with whiplash and 34 healthy controls (80% power, 5% significance, 0.53 effect size, one tailed test) would allow the detection of a 20% smaller difference in intraepidermal nerve fibre density in the acute stage compared to healthy controls based on our previous cohort of patients with chronic WAD2 (53).

Previous longitudinal studies on WAD2 have demonstrated a <15% dropout rate over six months (54, 55), and therefore we will recruit 115 participants with acute WAD2 and 34 healthy control participants.

Statistical analysis

Data will be exported to Stata (Version 17, StataCorp LLC, Texas, USA) for analysis. The distribution of the data will be checked for normality, and parametric, or non-parametric methods will be used as appropriate. Participants' characteristics will be described at all time points.

For objective 1, regression analysis, or non-parametric tests, will be used as appropriate to identify differences in MRI parameters (T2-weighted STIR 4 nerve roots per side, nerve morphology and DTI

For peer review only - http://bmjopen.bmj.com/site/about/guidelines.xhtml

parameters) and clinical tests (e.g., neuropathic pain scores, tests of nerve mechanosensitivity, QST and serum inflammatory markers) between acute WAD2 participants and age/gender-matched controls. Regression analysis will be used to assess associations with age and height.

For objective 2, recovery of participants with WAD2 will be determined at 6 months with the Neck Disability Index and a global recovery question. The analysis will be conducted with three models using different predictors measured in the acute stage: a) MRI parameters of neuroinflammation, b) clinical parameters that reflect neuroinflammation and c) the addition of MRI to clinical parameters to determine whether this improves predictive strength. The dependence between MRI and clinical assessments will be investigated and composite measures added to the model. In addition, a linear regression will be performed of Neck Disability Index scores on measures of neuroinflammation.

For objective 3, mixed effects modelling will be used to compare MRI and clinical measures between recovered and non-recovered participants with WAD2 over time.

For objective 4, multivariable regression will be used to investigate the relationship of each MRI variable with the clinical parameters of neuroinflammation. In addition, a regression analysis will be performed between clinical measures of neuroinflammation and MRI parameters.

For the sub-study, independent t-tests or nonparametric alternatives will be used to compare dermal and intraepidermal nerve fibre density in skin biopsies in patients in the acute phase after whiplash injury with healthy controls. Separate regression models will be used to investigate whether changes in nerve fibre density in the acute stage predict pain persistence at 6 months.

Data reduction techniques (e.g., principal components analysis) may be used prior to regression modelling.

Missing data will be managed with multiple imputation strategies.

Standardisation across the two sites

All clinical test procedures will be piloted between sites to ensure standardisation. Inter-rater reliability of range of motion using the inclinometer for nerve mechanical sensitivity testing will be performed. The German Network of Neuropathic Pain QST protocol has shown to have substantial inter-rater and inter-centre reliability (56, 57). The same MR scanners and coils are available at both sites so that settings will be identical. Any differences between sites will be adjusted at the analysis stage by comparing healthy controls at both sites.

Several quality control checks will be performed including checking of data entry (exchange of data sets between sites) and a comparison of healthy control data obtained at both sites.

For peer review only - http://bmjopen.bmj.com/site/about/guidelines.xhtml

Data sharing

Project data will be made available via open access publications, as well as the study webpage and the Universities of Sussex and Oxford repository, which are openly accessible. A statement will be placed on the study webpage and publications asking new users to contact the principal investigator if they wish to have access to the original data set. Anonymised data will be made accessible upon reasonable request once published or following completion of the study, adhering to data protection regulation and the Human Tissue Act (2004).

For peer review only

Current status of the study

Recruitment of participants to the study commenced in July 2021 and will continue until early summer 2023. The study is expected to be completed by August 2024.

ETHICS AND DISSEMINATION

The study has received sponsorship from the University of Sussex and ethical approval from the London-Brighton & Sussex Research Ethics Committee (20/PR/0625) and South Central – Oxford C Ethics Committee (18/SC/0263). The study is sponsored by University of Sussex which provides oversight for the totality of research sponsored by the University throughout the life-cycle of the study. Additionally, the study is independently reviewed every 6 months by the funding body. Any protocol amendments will be scrutinised and approved by both the study sponsor and the Health Research Authority and changes to the protocol amended at clinical trials.gov.

The results of this study should be of great interest to people after whiplash injury, clinicians, researchers, legal and insurance companies and policy makers. Dissemination via a multitude of platforms is therefore necessary to enhance its reach. We plan to publish traditionally in open access peer reviewed journals as well as presenting at national and international conferences. In addition, dissemination will occur via social media outlets. Patient and clinician information resources such as infographics will be produced and disseminated. Additionally, the patient partner group will support best strategies for dissemination to reach patient groups as well as policy makers. Local presentations to stakeholder groups such as patient groups, health authorities and professional bodies will be given.

For peer review only - http://bmjopen.bmj.com/site/about/guidelines.xhtml

Contributors

AD, AS and JG conceptualised the study. CR, JF, JG, AS, AD wrote the manuscript, SB oversaw the statistical aspects of the protocol and MC determined MRI sequence and analyses. All authors (CR, JF, AD, AS, JG, SB, MC, CT, AN, RY) reviewed drafts of the manuscript and approved the final version.

Funding

This work is supported by a Pain Challenge Grant from Versus Arthritis (number [22465]). ABS is supported by a Wellcome Trust Clinical Career Development Fellowship (222101/Z/20/Z) and the Medical Research Foundation (MRF-160-0013-ELP-SCHM-C0842). Her research is supported by the National Institute for Health Research (NIHR) Oxford Biomedical Research Centre (BRC). The views expressed are those of the authors and not necessarily those of the NHS, the NIHR or the Department of Health. This research was funded in whole, or in part, by the Wellcome Trust. For the purpose of Open Access, the author has applied a CC BY public copyright license to any Author Accepted Manuscript version arising from this submission.

Competing interests

There are no competing interests from the authors.

For peer review only

REFERENCES

1. Joslin CC, Khan SN, Bannister GC. Long-term disability after neck injury. a comparative study. J Bone Joint Surg Br. 2004;86(7):1032-4.
2. Spitzer WO, Skovron ML, Salmi LR, Cassidy JD, Duranceau J, Suissa S, et al. Scientific monograph of the Quebec Task Force on Whiplash-Associated Disorders: redefining "whiplash" and its management. Spine (Phila Pa 1976). 1995;20(8 Suppl):1S-73S.
3. Tournier C, Hours M, Charnay P, Chossegros L, Tardy H. Five years after the accident, whiplash casualties still have poorer quality of life in the physical domain than other mildly injured casualties: analysis of the ESPARR cohort. BMC Public Health. 2016;16:13.
4. Commission MA. Clinical guidelines for best practice management of acute and chronic whiplash-associated disorders. 2008.
5. Walton DM, Macdermid JC, Giorgianni AA, Mascarenhas JC, West SC, Zammit CA. Risk factors for persistent problems following acute whiplash injury: update of a systematic review and meta-analysis. J Orthop Sports Phys Ther. 2013;43(2):31-43.
6. Rushton A, Wright C, Heneghan N, Eveleigh G, Calvert M, Freemantle N. Physiotherapy rehabilitation for whiplash associated disorder II: a systematic review and meta-analysis of randomised controlled trials. BMJ Open. 2011(1):e000265.
7. Teasell R, McClure J, Walton D, Pretty J, Salter K, Meyer M, et al. A research synthesis of therapeutic interventions for whiplash-associated disorder (WAD): Part 4 – noninvasive interventions for chronic WAD. Pain Research & Management. 2010;15(5):313-22.

For peer review only - http://bmjopen.bmj.com/site/about/guidelines.xhtml

8. Greening J, Dilley A, Lynn B. In vivo study of nerve movement and mechanosensitivity of the median nerve in whiplash and non-specific arm pain patients. Pain. 2005;115(3):248-53.
9. Greening J, Anantharaman K, Young R, Dilley A. Evidence for Increased Magnetic Resonance Imaging Signal Intensity and Morphological Changes in the Brachial Plexus and Median Nerves of Patients With Chronic Arm and Neck Pain Following Whiplash Injury. J Orthop Sports Phys Ther. 2018;48(7):523-32.
10. Chien A, Eliav E, Sterling M. Hypoaesthesia occurs with sensory hypersensitivity in chronic whiplash--further evidence of a neuropathic condition. Man Ther. 2009;14(2):138-46.
11. Chien A, Eliav E, Sterling M. Hypoesthesia occurs in acute whiplash irrespective of pain and disability levels and the presence of sensory hypersensitivity. Clin J Pain. 2008;24(9):759-66.
12. Sterling M, Jull G, Vicenzino B, Kenardy J. Sensory hypersensitivity occurs soon after whiplash injury and is associated with poor recovery. Pain. 2003;104(3):509-17.
13. Moog M, Quintner J, Hall T, Zusman M. The late whiplash syndrome: a psychophysical study. Eur J Pain. 2002;6(4):283-94.
14. Fundaun J, Kolski M, Baskozos G, Dilley A, Sterling M, Schmid AB. Nerve pathology and neuropathic pain after whiplash injury: a systematic review and meta-analysis. Pain. 2021.
15. Pulman KG, Smith M, Mengozzi M, Ghezzi P, Dilley A. The erythropoietin-derived peptide ARA290 reverses mechanical allodynia in the neuritis model. Neuroscience. 2013;233:174-83.
16. Satkeviciute I, Dilley A. [EXPRESS] Neuritis and vinblastine-induced axonal transport disruption lead to signs of altered dorsal horn excitability. Mol Pain. 2018:1744806918799581.
17. Bove GM, Ransil BJ, Lin HC, Leem JG. Inflammation induces ectopic mechanical sensitivity in axons of nociceptors innervating deep tissues. JNeurophysiol. 2003;90(3):1949-55.
18. Dilley A, Lynn B, Pang SJ. Pressure and stretch mechanosensitivity of peripheral nerve fibres following local inflammation of the nerve trunk. Pain. 2005;117(3):462-72.
19. Goodwin G, Bove GM, Dayment B, Dilley A. Characterizing the Mechanical Properties of Ectopic Axonal Receptive Fields in Inflamed Nerves and Following Axonal Transport Disruption. Neuroscience. 2020;429:10-22.
20. Dilley A, Bove GM. Resolution of inflammation induced axonal mechanical sensitivity and conduction slowing in C-fiber nociceptors. Journal of Pain. 2008;9(2):185-92.
21. Filler AG, Howe FA, Hayes CE, Kliot M, Winn HR, Bell, et al. Magnetic resonance neurography. Lancet. 1993;341(8846):659-61.
22. Schwarz D, Kele H, Kronlage M, Godel T, Hilgenfeld T, Bendszus M, et al. Diagnostic Value of Magnetic Resonance Neurography in Cervical Radiculopathy: Plexus Patterns and Peripheral Nerve Lesions. Invest Radiol. 2018;53(3):158-66.
23. Baumer P, Dombert T, Staub F, Kaestel T, Bartsch AJ, Heiland S, et al. Ulnar Neuropathy at the Elbow: MR Neurography--Nerve T2 Signal Increase and Caliber. Radiology. 2011;260(1):199-206.
24. Boretius S, Gadjanski I, Demmer I, Bahr M, Diem R, Michaelis T, et al. MRI of optic neuritis in a rat model. Neuroimage. 2008;41(2):323-34.
25. Stanisz GJ, Webb S, Munro CA, Pun T, Midha R. MR properties of excised neural tissue following experimentally induced inflammation. Magnetic Resonance in Medicine. 2004;51(3):473-9.
26. Schmid AB, Campbell J, Hurley SA, Jbabdi S, Andersson JL, Jenkinson M, et al. Feasibility of Diffusion Tensor and Morphologic Imaging of Peripheral Nerves at Ultra-High Field Strength. Invest Radiol. 2018;53(12):705-13.
27. Guggenberger R, Eppenberger P, Markovic D, Nanz D, Chhabra A, Pruessmann KP, et al. MR neurography of the median nerve at 3.0T: optimization of diffusion tensor imaging and fiber tractography. Eur J Radiol. 2012;81(7):e775-82.
28. Heckel A, Weiler M, Xia A, Ruetters M, Pham M, Bendszus M, et al. Peripheral Nerve Diffusion Tensor Imaging: Assessment of Axon and Myelin Sheath Integrity. PLoS One. 2015;10(6):e0130833.

For peer review only - http://bmjopen.bmj.com/site/about/guidelines.xhtml

29. Song SK, Sun SW, Ju WK, Lin SJ, Cross AH, Neufeld AH. Diffusion tensor imaging detects and differentiates axon and myelin degeneration in mouse optic nerve after retinal ischemia. Neuroimage. 2003;20(3):1714-22.
30. Lin M, He H, Schifitto G, Zhong J. Simulation of changes in diffusion related to different pathologies at cellular level after traumatic brain injury. Magn Reson Med. 2016;76(1):290-300.
31. Vernon H, Mior S. The Neck Disability Index: a study of reliability and validity. J Manipulative Physiol Ther. 1991;14(7):409-15.
32. Vernon H. The Neck Disability Index: state-of-the-art, 1991-2008. J Manipulative Physiol Ther. 2008;31(7):491-502.
33. Freynhagen R, Baron R, Gockel U, Tolle TR. painDETECT: a new screening questionnaire to identify neuropathic components in patients with back pain. Curr Med Res Opin. 2006;22(10):1911-20.
34. Hansen M, Andersen TE, Armour C, Elklit A, Palic S, Mackrill T. PTSD-8: A Short PTSD Inventory. Clin Pract Epidemiol Ment Health. 2010;6:101-8.
35. Osman A, Barrios FX, Kopper BA, Hauptmann W, Jones J, O'Neill E. Factor structure, reliability, and validity of the Pain Catastrophizing Scale. J Behav Med. 1997;20(6):589-605.
36. Lovibond PF, Lovibond SH. The structure of negative emotional states: comparison of the Depression Anxiety Stress Scales (DASS) with the Beck Depression and Anxiety Inventories. Behav Res Ther. 1995;33(3):335-43.
37. Herdman M, Gudex C, Lloyd A, Janssen M, Kind P, Parkin D, et al. Development and preliminary testing of the new five-level version of EQ-5D (EQ-5D-5L). Qual Life Res. 2011;20(10):1727-36.
38. Creamer M, Bell R, Failla S. Psychometric properties of the Impact of Event Scale - Revised. Behav Res Ther. 2003;41(12):1489-96.
39. Nee RJ, Jull GA, Vicenzino B, Coppieters MW. The validity of upper-limb neurodynamic tests for detecting peripheral neuropathic pain. J Orthop Sports Phys Ther. 2012;42(5):413-24.
40. Butler D. The Sensitive nervous system: Noigroup Publications, 2000.
41. Kuschner SH, Ebramzadeh E, Johnson D, Brien WW, Sherman R. Tinel's sign and Phalen's test in carpal tunnel syndrome. Orthopedics. 1992;15(11):1297-302.
42. Rolke R, Baron R, Maier C, Tölle TR, Treede DR, Beyer A, et al. Quantitative sensory testing in the German Research Network on Neuropathic Pain (DFNS): standardized protocol and reference values. Pain. 2006;123(3):231-43.
43. Porter DA, Heidemann RM. High resolution diffusion-weighted imaging using readout-segmented echo-planar imaging, parallel imaging and a two-dimensional navigator-based reacquisition. Magn Reson Med. 2009;62(2):468-75.
44. Baskozos G, Sandy-Hindmarch O, Clark AJ, Windsor K, Karlsson P, Weir GA, et al. Molecular and cellular correlates of human nerve regeneration: ADCYAP1/PACAP enhance nerve outgrowth. Brain. 2020;143(7):2009-26.
45. Schmid AB, Bland JD, Bhat MA, Bennett DL. The relationship of nerve fibre pathology to sensory function in entrapment neuropathy. Brain. 2014;137(Pt 12):3186-99.
46. Lauria G, Hsieh ST, Johansson O, Kennedy WR, Leger JM, Mellgren SI, et al. European Federation of Neurological Societies/Peripheral Nerve Society Guideline on the use of skin biopsy in the diagnosis of small fiber neuropathy. Report of a joint task force of the European Federation of Neurological Societies and the Peripheral Nerve Society. Eur J Neurol. 2010;17(7):903-12, e44-9.
47. Doppler K, Werner C, Henneges C, Sommer C. Analysis of myelinated fibers in human skin biopsies of patients with neuropathies. J Neurol. 2012;259(9):1879-87.
48. Fadnavis S, Batson J, Garyfallidis E. Patch2Self: Denoising Diffusion MRI with Self-Supervised Learning. Proceedings of the 34th International Conference on Neural Information Processing Systems; Vancouver, Canada2020. p. 16293–303.
49. Garyfallidis E, Brett M, Amirbekian B, Rokem A, van der Walt S, Descoteaux M, et al. Dipy, a library for the analysis of diffusion MRI data. Front Neuroinform. 2014;8:8.

For peer review only - http://bmjopen.bmj.com/site/about/guidelines.xhtml

50. Veraart J, Fieremans E, Novikov DS. Diffusion MRI noise mapping using random matrix theory. Magn Reson Med. 2016;76(5):1582-93.
51. Carroll LJ, Holm LW, Hogg-Johnson S, Cote P, Cassidy JD, Haldeman S, et al. Course and prognostic factors for neck pain in whiplash-associated disorders (WAD): results of the Bone and Joint Decade 2000-2010 Task Force on Neck Pain and Its Associated Disorders. Spine (Phila Pa 1976). 2008;33(4 Suppl):S83-92.
52. VanVoorhis CRW, Morgan BL. Understanding Power and Rules of Thumb for Determining Sample Sizes. Tutorials in Quantitative Methods for Psychology. 2007;3(2):43-50.
53. Farrell SF, Sterling M, Irving-Rodgers H, Schmid AB. Small fibre pathology in chronic whiplash-associated disorder: A cross-sectional study. Eur J Pain. 2020;24(6):1045-57.
54. Sterling M, Elliott JM, Cabot PJ. The course of serum inflammatory biomarkers following whiplash injury and their relationship to sensory and muscle measures: a longitudinal cohort study. PLoS One. 2013;8(10):e77903.
55. Sterling M, Jull G, Vicenzino B, Kenardy J, Darnell R. Physical and psychological factors predict outcome following whiplash injury. Pain. 2005;114(1-2):141-8.
56. Middlebrook N, Heneghan NR, Evans DW, Rushton A, Falla D. Reliability of temporal summation, thermal and pressure pain thresholds in a healthy cohort and musculoskeletal trauma population. PloS one. 2020;15(5):e0233521-e.
57. Geber C, Klein T, Azad S, Birklein F, Gierthmühlen J, Huge V, et al. Test-retest and interobserver reliability of quantitative sensory testing according to the protocol of the German Research Network on Neuropathic Pain (DFNS): a multi-centre study. Pain. 2011;152(3):548-56.

FIGURE TITLE

Figure 1. Flow diagram showing the study design

For peer review only

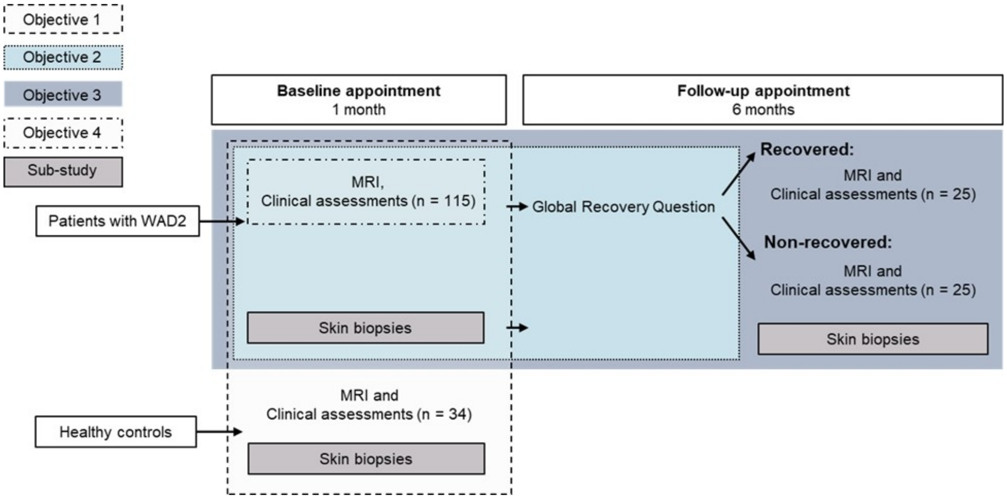

Figure 1. Flow diagram showing the study design

164x80mm (150 x 150 DPI)

SPIRIT

STANDARD PROTOCOL ITEMS: RECOMMENDATIONS FOR INTERVENTIONAL TRIALS

SPIRIT 2013 Checklist: Recommended items to address in a clinical trial protocol and related documents*

Section/item	ItemNo	Description	Y/N/N/A
**Administrative information**			
Title	1	Descriptive title identifying the study design, population, interventions, and, if applicable, trial acronym	Y
Trial registration	2a	Trial identifier and registry name. If not yet registered, name of intended registry	Y
	2b	All items from the World Health Organization Trial Registration Data Set	
Protocol version	3	Date and version identifier	Y
Funding	4	Sources and types of financial, material, and other support	Y
Roles and responsibilities	5a	Names, affiliations, and roles of protocol contributors	Y
	5b	Name and contact information for the trial sponsor	Y
	5c	Role of study sponsor and funders, if any, in study design; collection, management, analysis, and interpretation of data; writing of the report; and the decision to submit the report for publication, including whether they will have ultimate authority over any of these activities	Y
	5d	Composition, roles, and responsibilities of the coordinating centre, steering committee, endpoint adjudication committee, data management team, and other individuals or groups overseeing the trial, if applicable (see Item 21a for data monitoring committee)	N/A
**Introduction**			
Background and rationale	6a	Description of research question and justification for undertaking the trial, including summary of relevant studies (published and unpublished) examining benefits and harms for each intervention	Y

For peer review only

	6b	Explanation for choice of comparators	Y
Objectives	7	Specific objectives or hypotheses	Y
Trial design	8	Description of trial design including type of trial (eg, parallel group, crossover, factorial, single group), allocation ratio, and framework (eg, superiority, equivalence, noninferiority, exploratory)	Y
**Methods: Participants, interventions, and outcomes**			
Study setting	9	Description of study settings (eg, community clinic, academic hospital) and list of countries where data will be collected. Reference to where list of study sites can be obtained	Y
Eligibility criteria	10	Inclusion and exclusion criteria for participants. If applicable, eligibility criteria for study centres and individuals who will perform the interventions (eg, surgeons, psychotherapists)	Y
Interventions	11a	Interventions for each group with sufficient detail to allow replication, including how and when they will be administered	N/A
	11b	Criteria for discontinuing or modifying allocated interventions for a given trial participant (eg, drug dose change in response to harms, participant request, or improving/worsening disease)	N/A
	11c	Strategies to improve adherence to intervention protocols, and any procedures for monitoring adherence (eg, drug tablet return, laboratory tests)	N/A
	11d	Relevant concomitant care and interventions that are permitted or prohibited during the trial	N/A
Outcomes	12	Primary, secondary, and other outcomes, including the specific measurement variable (eg, systolic blood pressure), analysis metric (eg, change from baseline, final value, time to event), method of aggregation (eg, median, proportion), and time point for each outcome. Explanation of the clinical relevance of chosen efficacy and harm outcomes is strongly recommended	Y
Participant timeline	13	Time schedule of enrolment, interventions (including any run-ins and washouts), assessments, and visits for participants. A schematic diagram is highly recommended (see Figure)	Y

For peer review only - http://bmjopen.bmj.com/site/about/guidelines.xhtml

Sample size	14	Estimated number of participants needed to achieve study objectives and how it was determined, including clinical and statistical assumptions supporting any sample size calculations	Y
Recruitment	15	Strategies for achieving adequate participant enrolment to reach target sample size	Y

Methods: Assignment of interventions (for controlled trials)

Allocation:

Sequence generation	16a	Method of generating the allocation sequence (eg, computer-generated random numbers), and list of any factors for stratification. To reduce predictability of a random sequence, details of any planned restriction (eg, blocking) should be provided in a separate document that is unavailable to those who enrol participants or assign interventions	N/A
Allocation concealment mechanism	16b	Mechanism of implementing the allocation sequence (eg, central telephone; sequentially numbered, opaque, sealed envelopes), describing any steps to conceal the sequence until interventions are assigned	N/A
Implementati on	16c	Who will generate the allocation sequence, who will enrol participants, and who will assign participants to interventions	N/A
Blinding (masking)	17a	Who will be blinded after assignment to interventions (eg, trial participants, care providers, outcome assessors, data analysts), and how	N/A (blinding of data during analysis will take place).
	17b	If blinded, circumstances under which unblinding is permissible, and procedure for revealing a participant's allocated intervention during the trial	N/A

Methods: Data collection, management, and analysis

Data collection methods	18a	Plans for assessment and collection of outcome, baseline, and other trial data, including any related processes to promote data quality (eg, duplicate measurements, training of assessors) and a description of study instruments (eg, questionnaires, laboratory tests) along with their reliability and validity, if known. Reference to where data collection forms can be found, if not in the protocol	Y

For peer review only - http://bmjopen.bmj.com/site/about/guidelines.xhtml

	18b	Plans to promote participant retention and complete follow-up, including list of any outcome data to be collected for participants who discontinue or deviate from intervention protocols	Y
Data management	19	Plans for data entry, coding, security, and storage, including any related processes to promote data quality (eg, double data entry; range checks for data values). Reference to where details of data management procedures can be found, if not in the protocol	Y
Statistical methods	20a	Statistical methods for analysing primary and secondary outcomes. Reference to where other details of the statistical analysis plan can be found, if not in the protocol	Y
	20b	Methods for any additional analyses (eg, subgroup and adjusted analyses)	Y
	20c	Definition of analysis population relating to protocol non-adherence (eg, as randomised analysis), and any statistical methods to handle missing data (eg, multiple imputation)	Y
**Methods: Monitoring**			
Data monitoring	21a	Composition of data monitoring committee (DMC); summary of its role and reporting structure; statement of whether it is independent from the sponsor and competing interests; and reference to where further details about its charter can be found, if not in the protocol. Alternatively, an explanation of why a DMC is not needed	N/A Not an interventional trial
	21b	Description of any interim analyses and stopping guidelines, including who will have access to these interim results and make the final decision to terminate the trial	N/A
Harms	22	Plans for collecting, assessing, reporting, and managing solicited and spontaneously reported adverse events and other unintended effects of trial interventions or trial conduct	N/A
Auditing	23	Frequency and procedures for auditing trial conduct, if any, and whether the process will be independent from investigators and the sponsor	Y

For peer review only - http://bmjopen.bmj.com/site/about/guidelines.xhtml

Ethics and dissemination

Research ethics approval	24	Plans for seeking research ethics committee/institutional review board (REC/IRB) approval	Y
Protocol amendments	25	Plans for communicating important protocol modifications (eg, changes to eligibility criteria, outcomes, analyses) to relevant parties (eg, investigators, REC/IRBs, trial participants, trial registries, journals, regulators)	Y
Consent or assent	26a	Who will obtain informed consent or assent from potential trial participants or authorised surrogates, and how (see Item 32)	Y
	26b	Additional consent provisions for collection and use of participant data and biological specimens in ancillary studies, if applicable	Y
Confidentiality	27	How personal information about potential and enrolled participants will be collected, shared, and maintained in order to protect confidentiality before, during, and after the trial	Y
Declaration of interests	28	Financial and other competing interests for principal investigators for the overall trial and each study site	Y
Access to data	29	Statement of who will have access to the final trial dataset, and disclosure of contractual agreements that limit such access for investigators	N/A
Ancillary and post-trial care	30	Provisions, if any, for ancillary and post-trial care, and for compensation to those who suffer harm from trial participation	N/A
Dissemination policy	31a	Plans for investigators and sponsor to communicate trial results to participants, healthcare professionals, the public, and other relevant groups (eg, via publication, reporting in results databases, or other data sharing arrangements), including any publication restrictions	Y
	31b	Authorship eligibility guidelines and any intended use of professional writers	Y
	31c	Plans, if any, for granting public access to the full protocol, participant-level dataset, and statistical code	N/A

Appendices

For peer review only

Informed consent materials	32	Model consent form and other related documentation given to participants and authorised surrogates	N
Biological specimens	33	Plans for collection, laboratory evaluation, and storage of biological specimens for genetic or molecular analysis in the current trial and for future use in ancillary studies, if applicable	Y

*It is strongly recommended that this checklist be read in conjunction with the SPIRIT 2013 Explanation & Elaboration for important clarification on the items. Amendments to the protocol should be tracked and dated. The SPIRIT checklist is copyrighted by the SPIRIT Group under the Creative Commons "Attribution-NonCommercial-NoDerivs 3.0 Unported" license.

For peer review only
